# Absence of the highly expressed small carbohydrate-binding protein Cgt improves the acarbose formation in *Actinoplanes* sp. SE50/110

**DOI:** 10.1007/s00253-020-10584-1

**Published:** 2020-04-28

**Authors:** Lena Schaffert, Susanne Schneiker-Bekel, Jessica Gierhake, Julian Droste, Marcus Persicke, Winfried Rosen, Alfred Pühler, Jörn Kalinowski

**Affiliations:** 1 Microbial Genomics and Biotechnology, Center for Biotechnology, Bielefeld University, Sequenz 1, 33615 Bielefeld, Germany; 2 Product Supply, Bayer AG, Friedrich Ebert Str. 217-475, 42117 Wuppertal, Germany; 3 Senior Research Group in Genome Research of Industrial Microorganisms, Center for Biotechnology, Bielefeld University, Sequenz 1, 33615 Bielefeld, Germany

**Keywords:** Carbohydrate-binding module family 20 (CBM-20), Starch-binding assay, Carbon source utilization, CRISPR/Cas9

## Abstract

*Actinoplanes* sp. SE50/110 (ATCC 31044) is the wild type of industrial producer strains of acarbose. Acarbose has been used since the early 1990s as an inhibitor of intestinal human α-glucosidases in the medical treatment of type II diabetes mellitus. The small secreted protein Cgt, which consists of a single carbohydrate-binding module (CBM) 20-domain, was found to be highly expressed in *Actinoplanes* sp. SE50/110 in previous studies, but neither its function nor a possible role in the acarbose formation was explored, yet. Here, we demonstrated the starch-binding function of the Cgt protein in a binding assay. Transcription analysis showed that the *cgt* gene was strongly repressed in the presence of glucose or lactose. Due to this and its high abundance in the extracellular proteome of *Actinoplanes*, a functional role within the sugar metabolism or in the environmental stress protection was assumed. However, the gene deletion mutant ∆*cgt*, constructed by CRISPR/Cas9 technology, displayed no apparent phenotype in screening experiments testing for pH and osmolality stress, limited carbon source starch, and the excess of seven different sugars in liquid culture and further 97 carbon sources in the Omnilog Phenotypic Microarray System of Biolog. Therefore, a protective function as a surface protein or a function within the retainment and the utilization of carbon sources could not be experimentally validated. Remarkably, enhanced production of acarbose was determined yielding into 8–16% higher product titers when grown in maltose-containing medium.

**Electronic supplementary material:**

The online version of this article (10.1007/s00253-020-10584-1) contains supplementary material, which is available to authorized users.

## Introduction

Carbohydrate-binding modules (CBM) are widely distributed among proteins from all domains of life. They are involved in several processes and mechanisms, like carbohydrate metabolism, structure, energy storage, antibiosis, and immunological recognition (Guillén et al. 2010). CBMs often operate as auxiliary non-catalytic domains of protein complexes and enzymes. Here, they are combined with other (catalytic) domains and interact specifically to a certain substrate. Their function is bringing the biocatalysts into the direct vicinity of a substrate, enhancing the hydrolysis in case of insoluble substrates, disrupting the polysaccharide structures, and/or serving as protein anchor on the cell surface (Guillén et al. 2010).

CBMs also occur as single-domain proteins in prokaryotes and eukaryotes: In the Gram-negative bacterium *Serratia marcescens*, the non-catalytic chitin binding protein CBP-21 binds to insoluble crystalline substrates leading to structural changes, which increase substrate accessibility and enhance chitinolytic activity (Vaaje-Kolstad et al. 2005). Similarly, a single-domain CBM-33 protein has shown to enhance the activity of host cellulases in the Gram-positive actinomycete *Thermobifida fusca*, but this effect was only apparent at very low concentrations of cellulases and/or very long reaction times (Moser et al. 2008). In eukaryotes, the only described single-domain CBM is from olive pollen Ole e10, where it is potentially involved in the cell wall re-formation during germination (Barral et al. 2005).

There are three types of seven CBM fold families, which can be furthermore divided into 55 sub-families. These three types are classified according to structural and functional similarities into (A) surface-binding, (B) glycan-chain-binding, and (C) small sugar-binding (Guillén et al. 2010; Boraston et al. 2004).

A small protein consisting of a single CBM domain was found to be highly expressed in the Gram-positive soil-bacterium *Actinoplanes* sp. SE50/110 according to comprehensive studies of the extracellular proteome (Wendler et al. 2013; Ortseifen 2016) and transcriptome (Schwientek et al. 2013).

*Actinoplanes* sp. SE50/110 (ATCC 31044) is a slow-growing, aerobic actinomycete, which was originally isolated close to a coffee plantation in Kenia (Frommer et al. 1973). It is the wild type of industrial acarbose producer strains (Wehmeier and Piepersberg 2004). Acarbose (acarviosyl-1,2-maltose) is a pseudo-tetrasaccharide, which is used since the early 1990s as an inhibitor of human intestinal α-glucosidases in the medical treatment of type II diabetes mellitus and marketed under the brand name Glucobay® (Wehmeier and Piepersberg 2004; Wehmeier and Piepersberg 2009). Due to its industrial relevance, *Actinoplanes* sp. SE50/110 was extensively studied in the last decades. Fundamental knowledge about the complete genome (Schwientek et al. 2012; Wolf et al. 2017b), transcriptome (Schwientek et al. 2013; Schwientek et al. 2014), proteome (Wendler et al. 2013, Wendler et al. 2015a, b; Ortseifen 2016), and acarbose-related metabolites (Wendler et al. 2014; Ortseifen 2016) was gained, leading to novel interesting targets, like Cgt.

The small carbohydrate-binding protein found in *Actinoplanes* sp. SE50/110 contains 149 amino acids and was named Cgt according to its high similarity to the C-terminal domain of cyclodextrin glycosyltransferases (Schwientek et al. 2013).

Two transmembrane segments were predicted in Cgt by analyses of Wendler et al. (2015a), of which one is located within the N-terminal signal peptide. The second was assumed to be required for a multimerization (Wendler et al. 2015a). Hints for such oligomerization were given by the pattern in 2D-gel proteome analysis (Ortseifen 2016; Wendler et al. 2013) as well as by the fact that Cgt was completely absent from the proteome of the enriched membrane and membrane shaving fraction (Wendler et al. 2015a). Therefore, it was classified as a true secreted protein, which is not directly anchored to the membrane (Wendler et al. 2015a). For Cgt, strong expression on maltose and weak expression on glucose were observed on transcript and protein level (Schwientek et al. 2013; Wolf 2017; Wendler et al. 2013).

Due to its extraordinary high abundance in the extracellular proteome and exceeding transcription together with other proteins of the carbohydrate metabolism, a function within the maltose/maltodextrin/starch metabolism was assumed (Schwientek et al. 2013; Wendler et al. 2013; Ortseifen 2016). Cgt was also suggested to act as surface protein for surface enlargement (Ortseifen 2016).

Here, we analyze the effect of different carbon sources on the expression of the *cgt* gene and confirm a starch-binding function in an in vitro assay. By gene deletion, effects on growth and acarbose production were examined. Furthermore, phenotypical effects concerning different carbon sources, osmolality, and pH were investigated.

## Materials and methods

### Bioinformatics

The distribution of singular CBM-20 domain proteins was analyzed by BlastP analyses using the National Center for Biotechnology Information (NCBI) non-redundant protein database (Altschul et al. 2005; Altschul et al. 1990). Since CBM-20 domains occur in a variety of different proteins and enzymes, data filtering had to be performed: Of the initial 3316 BlastP hits, all hits of eukaryotic origin and all enzymes with function-specific annotation or sizes above 350 amino acids were excluded. The domain structures of the remaining 80 BlastP hits were analyzed manually (Marchler-Bauer et al. 2010; Marchler-Bauer et al. 2015, 2017; Marchler-Bauer and Bryant 2004). Fifty-three contain two CBM-20 domains traversed by a glyco-hydro-77-superfamiliy 4-alpha-glucanotransferase domain. Ten contain additional domains, like alpha-amylase inhibitor domains (a total of 5), N-terminal CBM-25 (a total of 2), CBM-26 (a total of 1) and IPT-superfamily domains with probable regulatory function (a total of 2) and a DUF1393-domain (a total of 1), which was described to occur in several alpha-amylases (information taken from the NCBI database). These candidates were also excluded. Only 18 candidates (including Cgt from *Actinoplanes* sp. SE50/110) displayed a singular CBM-20 domain. A protein tree was created by Blast tree view 1.17.5 of the NCBI database on basis of a multiple sequence alignment performed by BlastP (Altschul et al. 1990; Altschul et al. 2005).

### Media and growth conditions of *Actinoplanes* sp. SE50/110

#### Preparation of spore solutions of *Actinoplanes* sp. SE50/110

Glycerol stocks were prepared from NBS-grown cultures, like described by Schaffert et al. (2019a). For spore formation, 200 to 300 μL of a glycerol stock was grown on agar plates of soy flour medium (SFM-agar) (20 g L^−1^ soy flour (SOBO® Naturkost, Cologne, Germany), 20 g L^−1^ D-mannitol, 20 g L^−1^ Bacto™ agar (Becton-Dickinson, Heidelberg, Germany), 167 μL 10 N NaOH in tap water). Spores could be harvested after 5–7 days of incubation at 28 °C by washing them off in 3 mL distilled water with a cotton swab.

#### Preparation of media

The complex medium NBS consists of 11 g L^−1^ glucose × 1 H_2_O, 4 g L^−1^ peptone, 4 g L^−1^ yeast extract, 1 g L^−1^ MgSO_4_·7H_2_O, 2 g L^−1^ KH_2_PO_4_, and 4 g L^−1^ K_2_HPO_4_.

Maltose minimal medium is composed of 72.06 g L^−1^ maltose·1H_2_O, 5 g L^−1^ (NH_4_)_2_SO_4_, 0.184 g L^−1^ FeCl_2_·4H_2_O, 5.7 g L^−1^ Na_3_C_6_H_5_O_7_·2H_2_O, 1 g L^−1^ MgCl_2_·6H_2_O, 2 g L^−1^ CaCl_2_·2H_2_O, trace elements (final concentration: 1 μM CuCl_2_, 50 μM ZnCl_2_, 7.5 μM MnCl_2_), and phosphate buffer consisting of 5 g L^−1^ each K_2_HPO_4_ and KH_2_PO_4_ in aqua distilled. For media preparation and filter sterilization, the protocols of Wendler et al. were followed (Wendler et al. 2013; Wendler et al. 2015a, b).

For C-molar substitution of the carbon source, 79.2 g L^−1^ glucose·1H_2_O, 71.9 g L^−1^ galactose, 68.4 g L^−1^ cellobiose, 72 g L^−1^ C-pur (*Cerestar* 01908, Cerestar GmbH, Krefeld, Germany), 71.9 g L^−1^ D-arabinose, or 72.0 g L^−1^ D-lactose were used instead of maltose monohydrate. To approach natural carbon sources of the soil bacterium, a 4% (w/v) opalescent solution of “starch soluble” from *Acros Organics* (part of Thermo Fisher Scientific, Geel, Belgium) was generated by preheating sterile water to 90 °C in a water bath and adding the weighed portion with stirring. To allow comparison of growth, a maltose minimal medium was created, in which the C-molarity should approximate the one in the starch medium (here net weight of 44.4 g L^−1^ maltose·1H_2_O used).

Minimal media with 1 g L^−1^, 2 g L^−1^, 3 g L^−1^, 4 g L^−1^, and 5 g L^−1^ “starch soluble” from *Acros Organics* were created for cultivation under limited carbon source. Media of different pH and osmolality were created by addition of correcting agents (HCl and NaOH) or varying of the concentration of the carbon source maltose respectively and addition of inositol, which is not metabolized according to this study (Fig. S3, the exact weighing of maltose and inositol is shown in table S2).

The pH and osmolality of all media were determined by the pH-meter Calimatic of Knick GmbH (Berlin, Germany) and the Osmomat 3000 of Gonotec GmbH (Berlin, Germany) according to the manufacturer’s instructions.

#### Shake flask cultivation

Cultivation was performed in 250-mL Corning® Erlenmeyer baffled cell culture flasks at 28 °C and 140 rpm for seven days. For inoculation, a spore solution was prepared, in which the optical densities (OD) of different strains and mutants were adjusted to a similar value between OD_600_ of 4 to 6. One milliliter was used for inoculation of a 50-mL culture. Cell dry weights were determined by harvesting 2 × 1 mL of the cell suspension in weighed reaction tubes. The samples were centrifuged (14,000 *g*, 2 min). The supernatants were stored at − 20 °C for later analyses. The pellets were washed with deionized water and centrifuged again to remove the washing solvent. The cell pellets were dried for 1 day at 60–70 °C, like described by Wolf et al. (2017a).

#### Miniaturized cultivation in the BioLector system of m2p-labs GmbH (Baesweiler, Germany)

Comparative growth experiments were performed in a 1-mL reaction volume in a 48-well FlowerPlate covered by a gas-permeable sealing foil (m2p-labs GmbH, Baesweiler, Germany) and incubated for 1 week at 28 °C and 800 rpm in the RoboLector® system of m2p-labs. Growth was recorded by the backscatter signal. For determination of the final cell dry weights, 800 μL of each well was sampled in weighed reaction tubes (14,000 *g*, 2 min), washed with deionized water, and dried for 1 day at 60–70 °C, like described above. The final cell dry weights were used for growth comparison, with exception of the screening experiment under limited C-source starch, as here the final cell dry weights were outside of the effective range of the balance. In this case, the final backscatter signals were used for growth comparison. The supernatant was stored at − 20 °C for later analyses.

### Acarbose quantification from the supernatant by high-performance liquid chromatography

Supernatants of maltose-grown cultures of *Actinoplanes* ssp. were centrifuged (20,000 *g*, 2 min), mixed 1:5 with methanol by vortexing, and centrifuged again to remove precipitate (20,000 *g*, 2 min). The sample was transferred to high-performance liquid chromatography (HPLC) vials and analyzed in the HPLC system 1100 series of Agilent (G1312A Binary Pump Serial # DE43616357, G1329A ALS autosampler Serial # DE43613/10, G1315A DAD Serial # DE72002469). As stationary phase, the Hypersil APS-2 column (125 × 4 mm, 3 μm particle size) of Thermo Fisher Scientific Inc. (Waltham, Massachusetts, USA) was used, heated to 40 °C. As mobile phase, an isocratic flow of 1 mL min^−1^ 68% acetonitrile (ACN) (solvent B) and 32% phosphate buffer (0.62 g L^−1^ KH_2_PO_4_ and 0.38 g L^−1^ Na_2_HPO_4_·2H_2_O) (solvent A) was applied. Forty microliters of each sample was injected and separated in a 10 min run. Detection of acarbose was carried out with a DAD detector at 210 nm (reference 360 nm) and quantified from the peak areas of a calibration curve.

### Starch-binding assay

Proteins from the extracellular phase of *Actinoplanes* sp. SE50/110 grown in maltose minimal medium were concentrated by use of Amicon® Ultra 15-mL Centrifugal Filters 3K (Merck Millipore) and washed with 20 mM Tris-HCl (pH 7.5). The concentrate was taken up in 800 μL of 20 mM Tris-HCl (pH 7.5). A Bradford assay was performed (Roti®-Nanoquant, Carl Roth) for total protein quantification. In the starch-binding assay, 80 μg of the total protein raw extract was mixed with 200 μL 10%, 7.5%, 5%, 2.5%, 1%, and 0% (w/v) potato starch (VWR, LOT 171054105) in Tris-HCl buffer (20 mM, pH 7.5) and incubated for 1 h at 28 °C. The supernatant and starch fraction were separated by centrifugation. The starch fraction was washed three times in Tris-HCl buffer (20 mM, pH 7.5) and taken up in the same volume as the supernatant fraction. All 12 fractions were analyzed by SDS-PAGE.

### SDS-polyacrylamide gel electrophoresis

A 15% running gel was prepared (8 mL 30% acrylamide/0.8% bisacrylamide (Rotiphorese® Gel 30 (37.5:1), 2 mL Tris-HCl (3 M, pH 8.8), 160 μL SDS (10% (w/v)), 5.7 mL aqua distilled, 133 μL APS (10% (w/v)), and 13.3 μL TEMED (Bio-Rad, Munich, Germany)). For the 3.75% stacking gel, 500 μL 30% acrylamide/0.8% bisacrylamide (Rotiphorese® Gel 30 (37.5:1) were mixed with 500 μL Tris-HCl (1 M, pH 6.8), 40 μL SDS (10% (w/v)), 1.95 mL aqua distilled, 980 μL sucrose (60% (w/v)), 3.5 μL TEMED (Bio-Rad), and 35 μL APS (10% (w/v)). The SDS-polyacrylamide gel electrophoresis (SDS-PAGE) running buffer consists of 25 mM Tris-HCl, 192 mM glycine, and 0.1% (w/v) SDS. Twenty-four microliters of each sample was mixed with 6 μL PBJR (5×) (100 mM Tris-HCl (pH 6.8), 20% (v/v) glycerin, 4% (w/v) SDS, 200 mM DTT, 0.03% (w/v) bromophenol blue in aqua distilled) and incubated for 5 min at 95 °C. ExcelBand™ 3-color Regular Range Protein Marker (SMOBIO Technology, Inc., Hsinchu City, Taiwan, R.O.C) was used as molecular size marker. The gel was run at 80 V (constant) and 45 mA for approximately 45 min (pre-run) and afterwards with 15 mA per gel in the electrophoresis chamber Mini PROTEAN® Tetra Cell (Bio-Rad). Staining was performed in 2 g L^−1^ Coomassie brilliant blue R250, 0.5 g L^−1^ Coomassie brilliant blue G250, 10% (v/v) glacial acetic acid, and 25% (v/v) isopropanol. The gel was washed with deionized water and destained twice for 15 min in 45% (v/v) ethanol and 10% (v/v) glacial acetic acid (quick destaining) and overnight in 7% (v/v) glacial acetic acid (slow destaining). The gels were scanned with the Microtek Scan Maker i800 (Microtek International Inc., Hsinchu, Taiwan).

### Matrix-assisted laser desorption ionization-time of flight-mass spectrometry

For matrix-assisted laser desorption ionization-time of flight-mass spectrometry (MALDI-TOF-MS) analysis, an in-gel protein digestion was performed following protocols of Hansmeier et al. (2006) and Wendler et al. (2013): The reaction tubes were washed several times in 60% (v/v) acetonitrile (ACN) and dried overnight to get rid of plasticizers. Excised protein bands were washed and destained twice in 30% (v/v) ACN with 0.1 M ammonium hydrogen carbonate for 10 min with gentle agitation. After drying of the gel-slices for 30 min in the Centrifugal Evaporator (SpeedVac) of Thermo Scientific (Waltham, MA, USA), 15 μL trypsin solution (10 mg mL^−1^ trypsin in 10 mM NH_4_HCO_3_) was added and incubated overnight at 37 °C. The gel-slices were dried again and rehydrated in 5–20 μL 50% (v/v) ACN with 0.1% TFA. The sample was stored at − 20 °C. For MALDI-TOF-MS, 1.5 μL of the sample was mixed with 1 μL Matrix (HCCA) solution (1 mg α-cyano-4-hydroxycinnamic acid dissolved in 50% (v/v) ACN) and spotted directly on the MTP Anchor ChipTM (Bruker Daltonics, Leipzig, Germany). MALDI mass spectra were obtained by use of the ultrafleXtreme mass spectrometer (Bruker Daltonics). The Mascot software (Perkins et al. 1999) was used with the following parameters: enzyme, trypsin; missed cleavages, 1; modifications, carbamidomethyl (C), and oxidation (M); peptide tolerance, ± 100 ppm; mass values, MH+, and monoisotopic. For automated protein identification, Mascot was searched against all protein coding sequences of the *Actinoplanes* sp. SE50/110 genome.

### Recombinant DNA work

#### Deletion of the gene *cgt* by CRISPR/Cas9 technique

For the construction of a Δ*cgt* (Δ*ACSP50_5024*) deletion mutant by CRISPR/Cas9 technique (clustered regular interspaced short palindromic repeats/CRISPR-associated endonuclease 9), the plasmid pCRISPomyces-2 was used (Cobb et al. 2015). The spacer sequence was selected according to Wolf et al. (2016) and ordered as oligonucleotides together with its reverse complement at metabion GmbH (Steinkirchen, Germany) (oligo_1: 5′-acgcAGCGTCGCCCGCTGGGAGAA-3′, oligo_2: 5′-aaacTTCTCCCAGCGGGCGACGCT-3′). The oligonucleotides were annealed to a double-strand and assembled with the plasmid by Golden Gate Assembly (Engler et al. 2008) by use of *Bsa*I (NEB, Ipswich, MA, USA) according to the protocol of Cobb et al. (2015). For repair of the Cas9-induced double-strand break, a deoxyribonucleic acid (DNA) template was cloned into the *Xba*I-linearized vector by Gibson Assembly (Gibson et al. 2009) according to a protocol of Schaffert et al. (2019a). As DNA template, flanking sequences up- and downstream of the target gene (each round about 1 kB) were amplified by a polymerase chain reaction (PCR) with the Phusion® High-Fidelity PCR Master Mix with GC Buffer (NEB, Ipswich, MA, USA) (Primer sequences: *cgt*_flank1_fw: 5′-tcggttgccgccgggcgttttttatCCGGTACCCTGCTCCTCGTC-3′, *cgt*_flank1_rv: 5′-gtgacgcattgacgcaggtcGAGGGATATGGCTCAGATAC-3′, *cgt*_flank2_fw: 5′-gtatctgagccatatccctcGACCTGCGTCAATGCGTCAC-3′, *cgt*_flank2_rv: 5′-gcggcctttttacggttcctggcctACCTGACCCTGCTGAAATGG-3′). The reaction mix was transferred to *Escherichia coli* DH5αMCR by chemical transformation according to a protocol of Beyer et al. (2015). Growth and selection of *E. coli* was performed by plating them on Luria/Miller broth (LB-media) with 15 g L^−1^ agar-agar KobeI (both: Carl Roth, GmbH&Co.KG, Karlsruhe, Germany) supplemented with 50 mg L^−1^ apramycin-sulfate. Plates were incubated for 10–14 h at 37 °C. Apramycin-resistant colonies were tested by PCR and gel electrophoresis as well as by Sanger sequencing by our in-house sequencing core facility (primer sequences for PCR: for: 5′-GGCGTTCCTGCAATTCTTAG-3′, rev: 5′-TCGCCACCTCTGACTTGAGC-3′; walking primer for sequencing: w1: 5′-CGCTGATCTTCAGCTTCC-3′, w2: 5′-GCCTTCACCTTCCATCTG-3′, w3: 5′-TCGGGAAAGCCGCCGGAG-3′).

### Conjugal transfer to *Actinoplanes* sp. SE50/110 and plasmid curing

Conjugation was performed with *E. coli* ET12567/pUZ8002 (Kieser 2000) as donor strain and competent *Actinoplanes* sp. SE50/110 cells according to a protocol of Schaffert et al. (2019a).

Plasmid curing was performed according to the protocol of Wolf et al. (2016). Exconjugants were tested for the deletion by PCR. The PCR-fragment was excised from the gel and sequenced by our in-house Sanger sequencing core facility (primer sequences: del_cgt_kon: 5′-GATCGGGTTCAGCAAAGC-3′, del_w2_cgt: 5′-GCCTTCACCTTCCATCTG-3′). Additionally, genomic DNA of the deletion mutant was sequenced by the MinION® of Oxford Nanopore (Oxford, UK) to exclude off-target effects by the CRISPR/Cas9 technique. For this, genomic DNA of an NBS-grown culture was isolated with the NucleoSpin® Microbial DNA Kit (Macherey-Nagel, Düren, Germany) and a library was prepared with help of the 1D Genomic DNA by ligation-Kit (Oxford Nanopore, Oxford, UK).

### Screening experiments in the Biolog® OmniLog Phenotypic Microarray System

Pre-screening experiments were performed in the Biolog® OmniLog Identification System (Hayward, CA, USA) to evaluate respiration on different carbon sources (panel PM1 and PM2). The wild type of *Actinoplanes* sp. SE50/110 and the deletion mutant ∆*cgt* were grown on SFM-agar plates, as described above. Cells were harvested by use of a sterile swab and diluted in the inoculating fluid IF-0a. The turbidity of the cell suspension was checked to achieve 80% transmittance in the turbidimeter of Biolog®, according to the manufacturer’s protocol. A total of 2.32 mL of the cell suspension was added to 20 mL IF-0a, 0.24 mL 0.5 M MgCl_2_, 0.24 mL 0.5 M Na_2_SO_4_, 0.24 mL 1.5 M NH_4_Cl, 0.24 mL 1.0 M Na_3_PO_4_, 0.24 mL aqua distilled, 0.24 mL Biolog redox dye mix G, and 0.24 mL metal ion cocktail (5.0 mM each: ZnCl_2_·7H_2_0, FeCl_2_·6H_2_O, MnCl_2_·4H_2_O, CaCl_2_·2H_2_O), according to the manufacturer’s protocol. The PM panels were inoculated with 100 μL per well of the prepared solution and incubated for 1 week in the OmniLog system (Mode 71000 Serial # 406) at 28–30 °C. Data evaluation was carried out with the manufacturer’s software (*Kinetic Analysis*, Biolog and *Omnilog 2.3*, Biolog).

### Sampling and RNA isolation

For transcript analysis, 2 × 1 mL samples from *Actinoplanes* cultures were taken during growth phase, separated from the supernatant by centrifugation (10 s) and snap-frozen in liquid nitrogen. Pellets were stored at − 80 °C until further processing. Cell disruption, RNA isolation, and digestion of DNA from frozen cell pellets were performed by use of 2-mL lysing matrix tubes (0.1 mm spherical silica beads, MP Biomedicals, Santa Ana, California, USA) and the NucleoSpin® RNA Plus kit in combination with the rDNAse Set (Macherey-Nagel, Düren, Germany) according to a protocol of Schaffert et al. (2019a). Residual DNA was tested negatively with two primer pairs binding to genomic DNA of *Actinoplanes* sp. SE50/110 and amplifying small fragments at round about 200–300 nt. The quantity of RNA was analyzed with the NanoDrop 1000 spectrometer (Peqlab, Erlangen, Germany).

### Reverse transcription quantitative PCR

Reverse transcription quantitative PCR was carried out according to the protocol of Wolf et al. (2017a) by use of the SensiFast SYBR No-Rox One-Step Kit (Bioline, London, UK) in 96-well lightcycler plates (Sarstedt, Nümbrecht, Germany) in a LightCycler 96 System of Roche (Mannheim, Germany). The relative RNA amount was normalized on total RNA (100 ng) and calculated as 2^−ΔCq^. ΔCq was calculated as the difference of the mean Cq in the mutant strain compared with the control strain. The primers are listed in Table 1.

**Table 1:** Primers used in qRT-PCR

Genetic locus	Fwd-primer (5′-3′)	Rev-primer (5′-3′)	Amplicon size (bp)
*cgt* (*ACSP50_5024*)	CACCACGTACTGGAACTC	GCGACCTTCAACGTGAC	192
*acbA* (*ACSP50_3609*)	TCATGCTCGGCGACAACCTG	GACCGGTTTCTCCTCGATGG	173
*acbB* (*ACSP50_3608*)	CCCGCTGCTCGAACAACTAC	CCGCCGATGTGATAGACCTC	205
*acbD* (*ACSP50_3611*)	ACGCCAACTACTGGATGGAC	TCGAGCGGTTGGTGTAGAAG	231
*acbE* (*ACSP50*_*3610*)	GCGCGGCATGAAGATCTACC	CGGACGGCTTCTCGAAGAAC	218
*acbV* (*ACSP50_3594*)	GCTTCCACGGCAAGACGATG	CGCTCACGTTGGGTTTCTC	196
*acbW* (*ACSP50_3593*)	GGTGTACGACCGGAACATGC	GTTCGGCGTGGATGTGGTTG	224
*acbZ* (*ACSP50*_*3590*)	CGGCAATTCGCTGTTCAGTG	TGTGCTTGACGGTGTCCATC	167

## Results

### Distribution of single-domain CBM-20 proteins in the eubacterial world

The singular CBM-20 domain protein Cgt is one of the most strongly expressed genes in *Actinoplanes* sp. SE50/110 and in derived acarbose producer strains (Ortseifen 2016; Wendler et al. 2015a; Schwientek et al. 2013). It is secreted via the Sec pathway according to SignalP-analysis (Almagro Armenteros et al. 2019) and makes up to 8% of the total secreted proteome of this organism (according to calculations performed with the unpublished data of Dr. S. Wendler). Cgt contains 149 amino acids and a CBM-20 domain of fold-family 1, functional group A, characterized by a β-sandwich structure (Schwientek et al. 2013; Guillén et al. 2010). Members of this family are described to bind starch (Guillén et al. 2010).

**Fig. 1:**
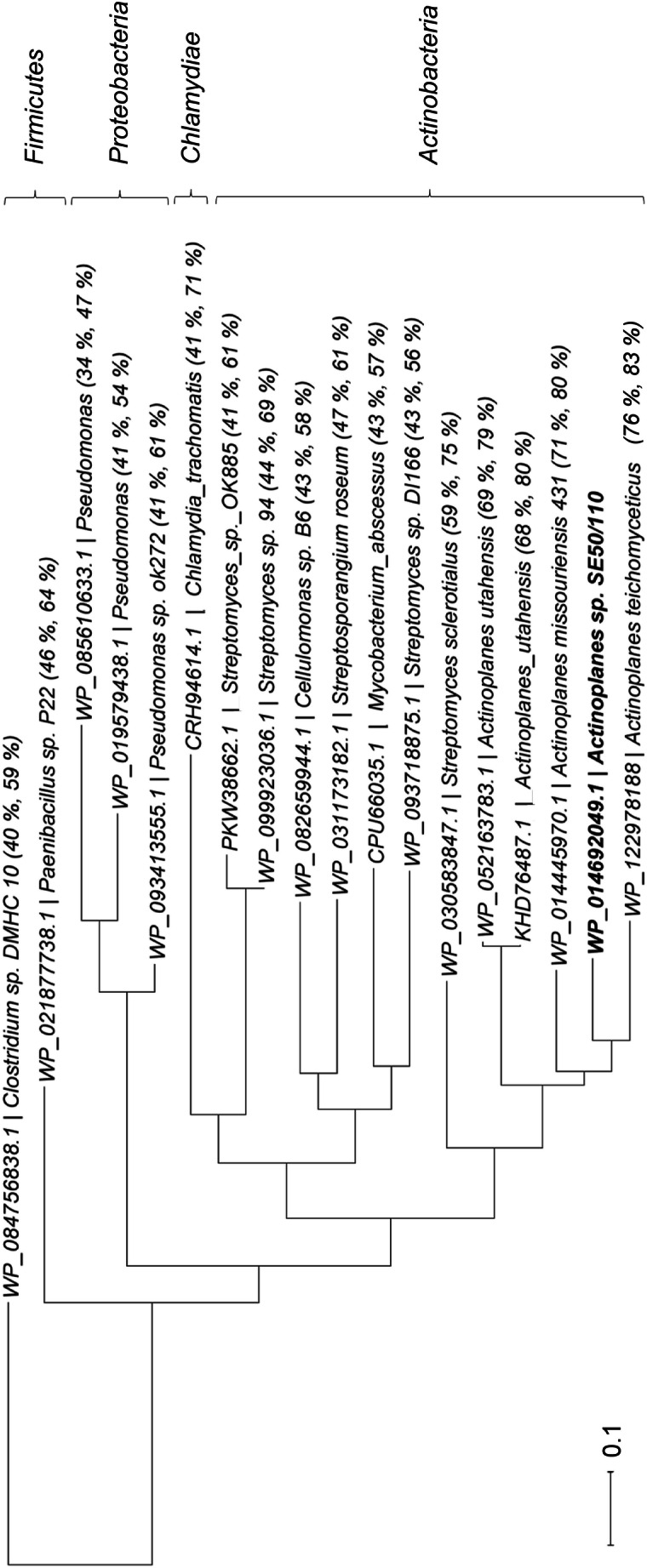
BlastP analysis of the amino acid sequence of Cgt leads to the identification of 17 other proteins consisting of a singular CBM-20 domain. The protein tree was created and visualized on the basis of a multiple sequence alignment performed by BlastP (Altschul et al. 2005; Altschul et al. 1990). The protein tree shows the distance of the 18 singular CBM-20 domain proteins, identified by their NCBI accession number and their hosts. The percentage of sequence identities and positives of BlastP analysis are shown in brackets

We analyzed the distribution of CBM-20 single-domain proteins in the prokaryotic world by BlastP analysis. Interestingly, singular CBM-20 domain proteins were found in only 17 other species (Fig. 1). Most of these occur in species of the order *Actinomycetales*, for example in strains of the genus *Actinoplanes*.

The majority of the 17 species were originally isolated from soil and environmental samples, namely *A. missouriensis* (Parenti and Coronelli 1979), *A. utahensis* (Parenti and Coronelli 1979; Couch 1963), *A. teichomyceticus* (Wink et al. 2006), *Streptomyces* sp. 94 (Chu et al. 1996), *Streptomyces* sp. OK885 (isolated from roots, Tennessee, USA, information taken from GenBank (NCBI database)), *Streptosporangium roseum* (Nolan et al. 2010), *Streptosporangium sclerotialus* (syn. *Chainia antibiotica*) (Thirumalachar 1955), *Cellulomonas* sp. B6 (Piccinni et al. 2016), *Paenibacillus* sp. P22 (Hanak et al. 2014), and *Clostridium* sp. DMHC 10, which was isolated from the sludge of a distillery waste treatment plant (Kamalaskar et al. 2010). CBM-20 proteins also occur in *Streptomyces* sp. DI166, for which the sampling sites has not been reported, and in multi-species of the family *Pseudomonadaceae*. These species belong to genera, which are known to include soil-inhabiting members.

Strains carrying singular CBM-20 proteins without direct connection to the habitats soil occur only occasionally, like in singular isolates of the human pathogens *Chlamydia trachomatis* (Thomson et al. 2008) and *Mycobacterium abscessus* (Ryan and Byrd 2018; Moore and Frerichs 1953).

In the case of the related species *Actinoplanes missouriensis*, the RNA-Seq data from the deletion mutant ∆*trcA* is publicly accessible at the DDBJ Sequence Read Archive (accession number DRA006277) (Mouri et al. 2018). According to this data, the Cgt-orthologue AMIS_58620 (WP_014445970.1) is massively transcribed in *A. missouriensis*, which is in accordance with the findings of SE50/110 (Ortseifen 2016; Wendler et al. 2015a; Schwientek et al. 2013).

### Confirmation of the starch-binding function by an in vitro assay

**Fig. 2:**
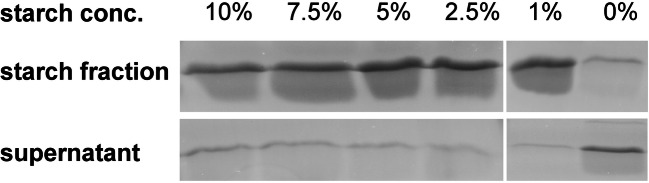
SDS-PAGE of the in vitro starch-binding assay. Shown are SDS-PAGE bands at round about 15 kDA, which correspond to Cgt (identified by MALDI-TOF-MS): In all starch-containing fractions, Cgt was detected. For the supernatant fractions, only slight bands were detected, indicating that Cgt has been nearly completely bound to the potato starch during the assay. In the negative control, most Cgt was found in the supernatant

CBM-20 domains are described to have a starch-binding function, which was tested by an in vitro assay in this work. As the small carbohydrate protein Cgt is highly expressed and secreted into the extracellular space due to an N-terminal signal peptide (Wendler et al. 2015a), the protein could be directly concentrated from the supernatant by filtration. A starch-binding assay was performed with potato starch in different concentrations. Both the starch fraction and the supernatant were analyzed by SDS-PAGE (Fig. 2, Fig. S1). In all starch fractions (ranging from 1 to 10% (w/v) of potato starch), a protein of a size of r. a. 15 kDA was detected, which was clearly identified as Cgt by MALDI-TOF-MS (Fig. 2, table S1). In contrast, the supernatant fractions were almost completely depleted by Cgt. In the negative control without starch, Cgt was mainly found in the supernatant fraction.

Besides Cgt, another small extracellular protein with starch-binding activity was identified by the assay (ACSP50_6253, data not shown).

### Analysis of *cgt* expression during growth on different carbon sources

The gene *cgt* has been reported of being differentially expressed in the presence of different carbon sources, which was determined by transcriptome and proteome analyses on glucose and maltose (Schwientek et al. 2013; Wendler et al.2015b; Ortseifen 2016).

Here, we tested the effects of several carbon sources on the expression of the *cgt* gene by measuring the transcript amounts by reverse transcription quantitative PCR (RT-qPCR). For this purpose, the wild type strain of *Actinoplanes* sp. SE50/110 was grown on minimal medium supplemented with maltose, glucose, starch, galactose, cellobiose, lactose, and C-Pur (*Cerestar* 01908) (Fig. S2). The latter is a sugar-containing product from the degradation of starch mainly consisting of maltose and maltotriose (Schaffert et al. 2019b). All carbon sources were supplemented in equivalent C-molar amounts. The only exception was starch: Due to the low solubility, a 4% (w/v) opalescent solution of “starch soluble” from *Acros Organics* was generated. For comparison, a maltose minimal medium with reduced amount of maltose was prepared (here, 44.40 g L^−1^ maltose monohydrate), in which the C-molarity should approximate the one in the starch medium.

**Fig. 3:**
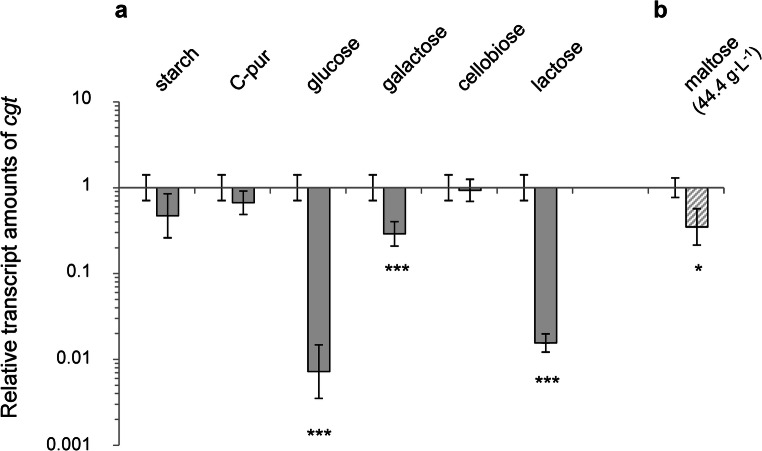
**a** Relative transcript amounts of *cgt* in *Actinoplanes* sp. SE50/110 grown on minimal medium supplemented with starch, C-Pur, glucose, galactose, cellobiose, or lactose as carbon source, compared with a culture grown on maltose minimal medium. Testing for differences in a two-sided *t* test displayed significant differential gene expression of *cgt* gene on the carbon sources glucose (*p* value = 0.002848), galactose (*p* value = 0.002945), and lactose (*p* value = 0.00114) compared with maltose. **b** Relative transcript amount of *cgt* in *Actinoplanes* sp. SE50/110 grown on maltose minimal medium complemented with 44.40 g L^−1^ maltose compared with a culture grown on 72.06 g L^−1^ maltose. Testing for differences in a two-sided *t* test displayed significant reduced gene expression of *cgt* in the medium containing reduced amounts of maltose (*p* value = 0.04141)

Differential transcription was observed for galactose to a minor extent (3.4-less transcribed, log_2_(fold-change) = 0.291) (Fig. 3a). A significant reduction of *cgt* transcription was measured for the carbon sources glucose (142-fold less transcribed, log_2_(fold-change) = 0.007) and lactose (62-fold less transcribed, log_2_(fold-change) = 0.016) (Fig. 3a).

When cells were grown on maltose minimal medium with reduced amount of maltose (here, 44.4 g L^−1^ instead of 72.06 g L^−1^), a 2.9-fold decreased transcription of the *cgt* gene was observed (log_2_(fold-change) = 0.345) (Fig. 3b). For the residual tested carbon sources, the transcription of the *cgt* gene was similar or just slightly and insignificantly reduced compared with a maltose-grown culture (Fig. 3a). Subsequently, we analyzed the growth behavior of a *cgt* deletion mutant on these carbon sources (see below).

### The gene deletion mutant ∆*cgt* has no apparent growth phenotype on different carbon sources or under carbon-limited conditions

The differential transcription profile of *cgt* (see above) indicates for a function within the sugar metabolism. Ortseifen (2016) suggested that Cgt might be responsible for the retention of carbon as energy source in the context of the carbophore model (Wehmeier and Piepersberg 2009). Due to this, we analyzed growth of the deletion mutant ∆*cgt* on different carbon sources.

A pre-screening experiment was performed in the OmniLog Phenotypic Microarray System (Biolog Inc., Hayward, USA), which allows fast phenotypic screening by measurement of the cellular respiration activity of a total of 190 different carbon sources. Here, *Actinoplanes* sp. SE50/110 displayed respiration on 103 carbon sources (Fig. S3). Except of arabinose and lactose, no differential respiration profile was observed for ∆*cgt* on 101 carbon sources. In order to validate these results on the level of growth, the carbon sources arabinose and lactose were tested in a shake flask cultivation together with maltose, glucose, and the complex carbon source starch. The disaccharide cellobiose were tested, too, to imitate a natural carbon source of the habitat soil. No restraints on growth were observed for ∆*cgt* on all tested carbon sources (Fig. S4 and S5), in spite of the differential expression of *cgt* on these carbon sources, like described before (see above).

Furthermore, growth under carbon-limited conditions with 1 g L^−1^, 2 g L^−1^, 3 g L^−1^, 4 g L^−1^, and 5 g L^−1^ starch was tested in the RoboLector®-system of m2p-labs. No disadvantages in growth were observed for the ∆*cgt* mutant compared with the wild type (Fig. S6).

### Cgt has no impact on osmolality- or pH-tolerance

Cgt multimers have been proposed to form surface layers through multimerization (Wendler et al. 2015a, b). Due to this, it has been assumed to fulfill a role in protection against environmental changes, like drought, pH, and osmolality.

A pH screening was performed on solid media as well as in liquid culture. For screening on solid media, SFM-agar plates of pH ranging from pH 4 to 11 (in steps of 1) were prepared and droplets of a dilution series of spores of the wild type and the deletion mutant ∆*cgt* were applied. Both mutant and wild type were able to grow from pH 5 to 11. No differences in growth or spore formation were observed (Fig. S7).

For pH screening in liquid culture in the RoboLector®-system, a 72 g L^−1^ maltose minimal medium of pH ranging from 4 to 7 (in steps of 0.5) was prepared. Higher pH values could not be tested in liquid culture, as medium components tend to precipitate. Both strains grew from pH 4.5 to 7, with ∆*cgt* displaying slightly accelerated growth at low pH (from 4.5 to 5.5) and slightly decelerated growth at neutral pH (from 6.0 to 7.0) (Fig. S8 and S9). However, regarding the final cell dry weights, these differences were not significant.

For osmolality screening, maltose minimal medium was prepared with different concentrations of maltose ranging from 3.6 to 108.1 g L^−1^ maltose monohydrate and osmolality ranging from 323.5 to 681.0 mOsmol kg^−1^ (S2 table). No significant growth differences were observed between the wild type and the deletion mutant ∆*cgt* (Fig. S10). Also, inositol was tested as osmolyte, since it is not consumed by *Actinoplanes* (Fig. S3). The osmolality ranged from 388.5 to 695.0 mOsmol kg ^−1^, but no growth differences were observed (Fig. S11). Lower osmolalities between 159 and 190 mOsmol kg^−1^ were tested by use of the complex medium NBS (Fig. S12, table S2). Again, no significant differences in growth were observed between the wild type strain and the deletion mutant ∆*cgt*.

Since an effect of drought tolerance is difficult to measure, we only accessed the colony and spore formation on the surface of the bacterial lawn and could not find any differences between the wild type and ∆*cgt*.

### The ∆*cgt* mutant displays an improved acarbose formation on maltose minimal medium

Although no distinct growth phenotype could be observed under the tested conditions described above, lack of the highly expressed Cgt protein was expected to save metabolic resources, such as ATP and amino acids. These might be used for cellular growth or other anabolic processes. In our experiments, ∆*cgt* has not displayed significant growth advantages. However, higher final acarbose concentrations were detected for the deletion mutant ∆*cgt* compared with the wild type (table S2). For the cultivation in complex medium, this was most striking during the growth phase (Fig. S12).

**Fig. 4:**
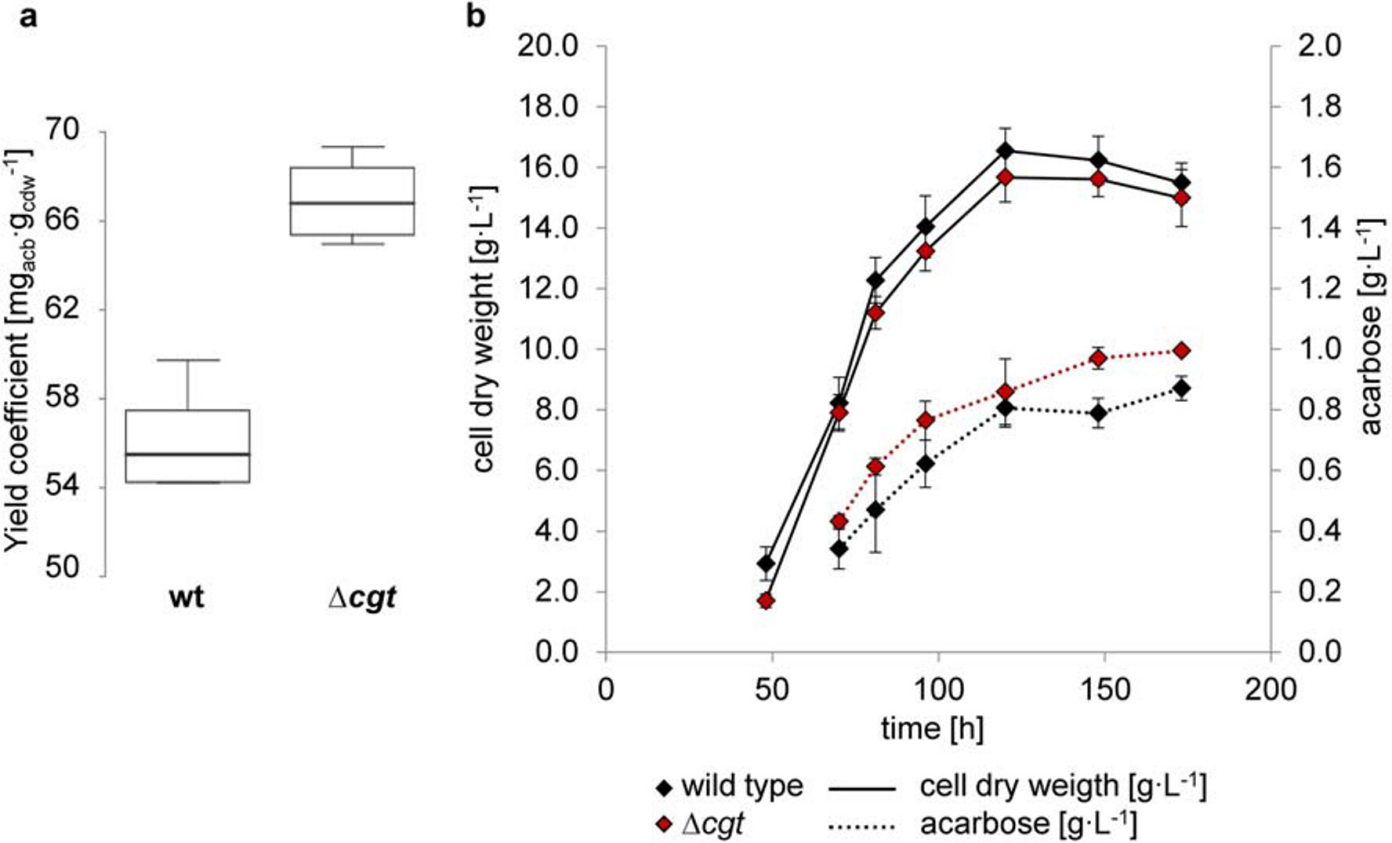
**a** Final yield coefficient of acarbose with reference to the cell dry weight in a box plot (created by the Interactive Dotplot tool (Weissgerber et al. 2017)). **b** Cell dry weights and acarbose concentrations in the supernatant during cultivation in maltose minimal medium (*n*_cdw_ = 5, *n*_acb_ = 4)

The improved acarbose-producing phenotype was validated by three independent shake flask cultivations in maltose minimal medium (Fig. 4, Fig. S13, S14, and table S3). Quantification of acarbose from the supernatant displayed an enhanced acarbose yield coefficient of the deletion mutant compared with the wild type. The differences in the final acarbose yields were significant (tested by a two-sided *t* test, *p* value = 0.04608). Thereby, in ∆*cgt*, an increase of 8.3 to 16.6% of the final acarbose concentration was achieved (table S3).

### Deletion of *cgt* has no impact on the expression of acarbose biosynthesis genes

**Fig. 5:**
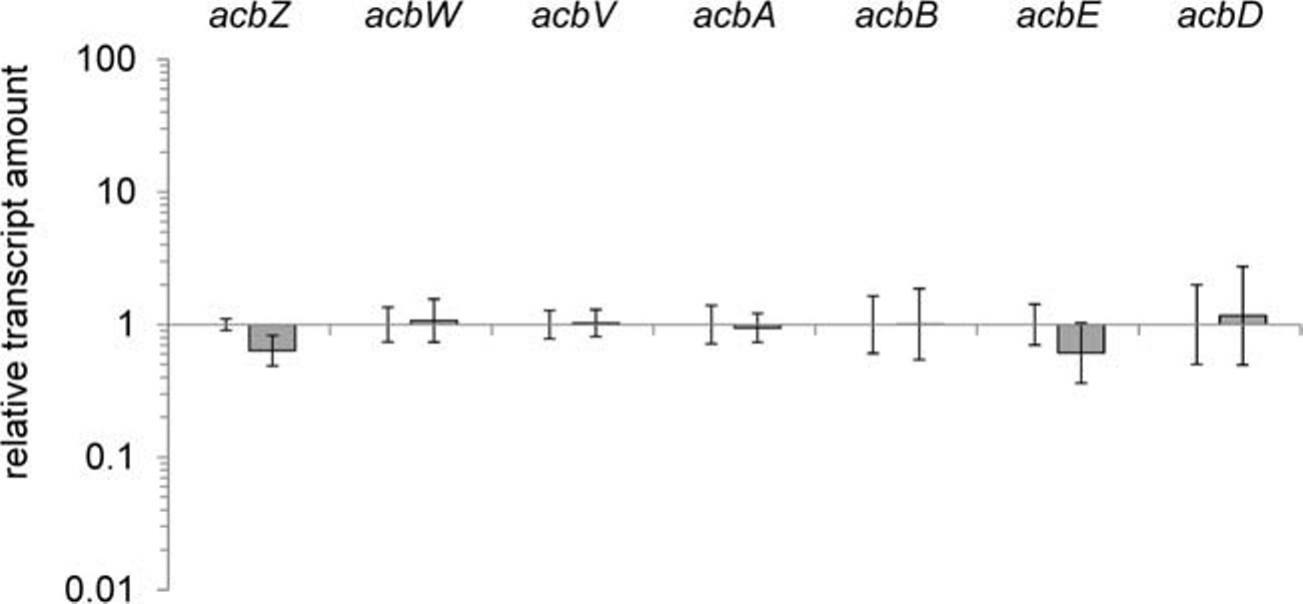
Relative transcript amounts of the genes *acbZ*, *acbW*, *acbV*, *acbA*, *acbB*, *acbE*, and *acbD* of the mutant ∆*cgt* compared with the wild type of *Actinoplanes* sp. SE50/110 grown on maltose minimal medium (with the number of biological replicates ranging between 3 and 6). The relative transcript amounts do not differ significantly

Findings that the deletion of the highly expressed gene *cgt* has no negative impact on growth or viability of the organism under various conditions, but yields into an enhanced acarbose-producing phenotype, were surprising. Due to this and to rule out a direct impact on the regulation of acarbose biosynthesis (*acb*) genes, RT-qPCRs of representative *acb* genes were performed. For this, the wild type and the deletion mutant ∆*cgt* were grown on maltose minimal medium and RNA was isolated from samples of the early growth phase. The relative transcript amount of the genes *acbZ*, *acbW*, *acbV*, *acbA*, *acbB*, *acbD*, and *acbE* were calculated for ∆*cgt* in comparison with the wild type (Fig. 5). The gene *acbV* is the first of several polycistronically transcribed genes within the main operon of the acarbose biosynthesis gene cluster (Wolf et al. 2017b). The monocistronically transcribed genes *acbD* and *acbE* encode proteins of the extracellular acarbose metabolism and have shown to be strongly regulated by the acarbose regulator AcrC (Wolf et al. 2017a). The genes *acbA*, *acbB*, and *acbZ* are monocistronically transcribed, too, and are annotated as enzymes of the acarbose biosynthesis (*acbAB*) respectively the extracellular sugar metabolism (*acbZ*). *AcbW* is the first gene of the *acbWXY*-operon, putatively encoding an ABC transporter. For all selected transcripts, no significant change in the relative transcript level was measured in the deletion mutant ∆*cgt* compared with the wild type (Fig. 5).

## Discussion

The connection of the carbohydrate metabolism and the acarbose biosynthesis is of highest interest, since recent research has pointed out the importance of carbon utilization in the context of the biosynthesis of acarbose and further acarviosyl-metabolites in the wild type (Wendler et al. 2014).

In this context, the starch-binding protein Cgt is striking. It is one of the strongest expressed genes in *Actinoplanes* sp. SE50/110 (Schwientek et al. 2013) making up for about 8% of the whole secreted proteome (unpublished data provided by Dr. Sergej Wendler). Its gene product is exported into the extracellular space (Wendler et al. 2013). Excess production and secretion means high costs for the cell: Just the translational process requires 4 ATP per peptide bond (Campbell and Reece 2011; Purves 2006), not including additional costs for RNA synthesis, amino acid production, protein folding, and export.

We therefore concluded that Cgt has a significant role in the physiology of *Actinoplanes* sp. SE50/110. Two different functions of Cgt were proposed and analyzed in this paper: A role within the sugar metabolism and a role as surface protein.

Due to the starch-binding domain, Ortseifen (2016) suggested that Cgt might be involved in binding and retention of energy sources in the context of the carbophore model (Wehmeier 2003). Evidence was also given in this work by RT-qPCR, which displayed differential expression of the gene *cgt* in glucose-, galactose-, and lactose-grown cultures compared with cultures grown on maltose, higher maltodextrins, and cellobiose. This is in accordance with differential proteome analyses on the carbon sources maltose and glucose (Wendler et al. 2015b). These results indicate a carbon-dependent expression of *cgt*. It would be exciting to elucidate the regulatory mechanism. However, it remains to be considered that over 900 genes are putatively involved in the transcriptional regulation of *Actinoplanes* sp. SE50/110, of which 697 are annotated as transcriptional regulators according to the annotation of Wolf et al. (2017b) (GenBank: LT827010.1). Since research on the regulatory network of *Actinoplanes* sp. SE50/110 is still in its early stages, we do not have any clue about the transcriptional regulation of *cgt*, yet.

A sugar-dependent expression of *cgt* might indicate a function within the utilization of maltose, higher maltodextrins, and—potentially—also cellobiose. However, our studies of the deletion mutant ∆*cgt* have not unveiled phenotypical differences regarding the carbon utilization. This was tested for a total of 105 different carbon sources, of which 103 were analyzed in the OmniLog screening system and six in a liquid culture.

As the function of Cgt might be negligible under excess of carbon source but indispensable when growing under conditions with limited carbon source, we tested growth of the deletion mutant ∆*cgt* and the wild type on minimal medium with low concentrations of starch. Starch was chosen as carbon source, due to the starch-binding activity of Cgt, which was confirmed in a starch-binding assay in this paper. Nevertheless, no differential growth phenotype could be observed under conditions of limited carbon source.

Another function within the sugar metabolism could consist in binding of insoluble crystalline substrates, which might lead to structural changes, that increases substrate accessibility and enhances the activity of other hydrolyzing enzymes, like amylases. Such mechanisms have already been described in the soil bacteria *Serratia marcescens* for chitinolysis (Vaaje-Kolstad et al. 2005) and *Thermobifida fusca* for cellulysis (Moser et al. 2008). In the genome of *Actinoplanes* sp. SE50/110, several genes are encoded with putative α-glycosidic function, of which three, the α-amylases/pullulanases AcbE, AcbZ, and PulA, were shown to accumulate in the extracellular space (Wendler et al. 2015a). Additionally, another small extracellular protein of unknown function and starch-binding capability (ACSP50_6253) was identified in our starch-binding assay. By heterologous expression of extracellular amylases and enzyme assays in presence and absence of Cgt and ACSP50_6253, a supporting function during starch degradation might be detected in future experiments. However, a study from the actinomycete *Thermobifida fusca* points out that such effects might be only apparent at very low concentrations of hydrolyzing enzyme and/or very long reaction times (Moser et al. 2008), which might make such proof of function difficult.

Apart from the sugar metabolism, also a function as surface layer protein is conceivable, which is supported by the fact that Cgt forms multimers (Ortseifen 2016; Wendler et al. 2013). Wendler et al. (2015a) identified two transmembrane domains in the Cgt protein, of which one is involved in translocation by the Sec pathway as part of the leader peptide and the second is assumed to be required for multimerization. Although Cgt is not likely to be physically anchored in the membrane (Wendler et al. 2015a), we assume that secreted Cgt proteins do not diffuse from the cell surface and remain as multimers in the mesh of the mycelium, because of the reduced volumetric flow rate in a mycelium. In this context, the starch-binding domain might serve as an anchor. As putative surface protein, it might have a protective function in the context of pH and osmolyte stress or drought.

However, our screening experiments showed that the deletion of *cgt* gene has not led to significant growth inhibitions at different pH in liquid culture. Tendencies for stalled growth for ∆*cgt* at neutral pH were certainly noted but turned out to be not significant. From the screening experiments on solid media, there was no indication that Cgt might have a protective function in case of pH or drought.

Hints for a putative function in the context of osmoregulation were given by reverse transcription quantitative PCR of the wild type, grown on different amounts of maltose. Here, we observed a 2.9-fold reduced transcription of the gene *cgt*, when growing on 44.4 g L^−1^ maltose (compared with a 72 g L^−1^), which might be an effect of osmolality. We analyzed growth of the deletion mutant ∆*cgt* in several screening experiments in liquid culture with osmolarities ranging from 159 to 681 mOsmol kg^−1^. Under all tested conditions, no differences in growth and viability were observed for the deletion mutant ∆*cgt* compared with the wild type.

As no apparent physiological impact was observed by the deletion of *cgt* gene neither in utilization of different carbon sources in excess nor in limitation, neither under different pH nor osmolyte conditions, it might be possible that the function of Cgt only becomes apparent in its natural environment and in possible competition with other soil organisms. Interestingly, we found similar independent singular CBM-20 domain proteins in 17 other prokaryotic species, most of which belong to the order *Actinomycetales*. Although rare, this at least displays a certain distribution and shows that Cgt is not a strain-specific protein. Most of the species harboring single-domain CBM-20 proteins were associated with soil habitats. Together with the fact that *cgt* is highly expressed in *Actinoplanes* sp. SE50/110 and in the related species *A. missouriensis* 431, this supports the hypothesis that proteins like Cgt fulfill a crucial function in bacteria living within this habitat. A function of Cgt in the direct contact with other microbial competitors could be tested by co-cultivations in future.

Although it was surprising that Cgt turned out to be dispensable under the tested laboratory conditions, we observed a positive phenotype regarding the acarbose production. An increase of the acarbose yield between 8.3 and 16.6% was achieved by deletion of *cgt*. Although the final product yields differ slightly between the batch cultivations, ∆*cgt* always performed significantly better than the wild type. This was shown in three independent shake flask and several micro-scale cultivations performed in maltose minimal medium over a time period of several month (data not shown). Thus, the improved producing phenotype was robust over long time periods and in different cultivation settings.

A direct regulatory effect by deletion of *cgt* on the expression of the *acb* genes was not observed. However, putative regulatory effects on post-transcriptional level cannot be excluded.

We assume that the improved producing phenotype is due to the metabolic burden by expression of *cgt* gene in the wild type, which brings relief of energy and of free resources in ∆*cgt*. The gene product of *cgt* is exported into the extracellular space (Wendler et al. 2013) making up for about 8% of the whole secreted proteome. Like already discussed, excess production and secretion are cost-intensive for the organism. Therefore, we assume that by deletion of cgt, energy and resources, such as ATP, are relieved. These resources are probably redirected to the acarbose biosynthesis, which is a strictly growth-associated product (Wolf et al. 2017a; Wendler et al. 2014).

By this, Cgt provides an example for the metabolic potential of *Actinoplanes* sp. SE50/110. Aside from the *acb* gene cluster, a total of 19 further secondary metabolite gene clusters were identified by an antiSMASH analysis (Wolf et al. 2017b). Some of these might be interesting targets for the future genome reduction, like the hybrid NRPS/PKS gene cluster cACPL_4 (*ACSP50_6119-6142*) (Schwientek et al. 2012). The genes of this biosynthesis gene cluster display an increased transcription during growth (Wolf 2017) and the proteins are localized at the inner membrane, together with the Acb proteins (Wendler et al. 2015b). Therefore, the deletion might bring relief to space and resources and improve the energy balance in producer strains of *Actinoplanes*. Since one-third of the coding sequence of SE50/110 is annotated as hypothetical or uncharacterized (Wolf et al. 2017a), there is a considerable potential for a further genome streamlining in future.

This work leaves some open questions about the role of the highly expressed small starch-binding protein Cgt in *Actinoplanes* sp. SE50/110. The next step is the transfer of this genetic deletion to further producer strains of *Actinoplanes*. Flux analyses and comparative RNA-Seq experiments of both different producer strains and the promising ∆*cgt* mutant might be carried out to gain further insights into the metabolic and genetic processes in *Actinoplanes* ssp. in future.

## Electronic supplementary material

ESM 1 (PDF 1.76 mb)
