# Supplementary material for: Absence of the highly expressed small carbohydrate-binding protein Cgt improves the acarbose formation in Actinoplanes sp. SE50/110
Source: Appl Microbiol Biotechnol. 2020 Apr 28;104(12):5395–408. doi: 10.1007/s00253-020-10584-1 (PMC7275007; doi:10.1007/s00253-020-10584-1)
Supplement: Supplementary file 1 — (PDF 1.76 mb) [file 253_2020_10584_MOESM1_ESM.pdf]

Supplementary material

Title of the article

Absence of the highly expressed small carbohydrate binding protein Cgt improves the acarbose formation in *Actinoplanes* sp. SE50/110

Journal name

Applied Microbiology and Biotechnology

Author names

Lena Schaffert[1¶], Susanne Schneiker-Bekel[1&], Jessica Gierhake[1&], Julian Droste[1&], Marcus Persicke[1&], Winfried Rosen[3&], Alfred Pühler[2&], Jörn Kalinowski[1&*]

Affiliation

[1] Microbial Genomics and Biotechnology,

[2] Senior Research Group in Genome Research of Industrial Microorganisms,

Center for Biotechnology, Bielefeld University, Sequenz 1, 33615 Bielefeld, Germany

[3] Product Supply, Bayer AG, Friedrich Ebert Str. 217-475, 42117 Wuppertal, Germany

[*] Corresponding author, Email: joern@cebitec.uni-bielefeld.de, Telephone: 0049 521 106008756

Fig. S1 SDS-PAGE of an *in vitro* starch binding assay. Bands at round about 15 kDA correspond to Cgt (identified by MALDI-TOF-MS, table S1): In all starch-containing fractions (left, ranging from 1 to 10 % (w/v) of starch), Cgt was detected. For the supernatant fractions (right) only slight bands were detected, indicating that Cgt has been nearly completely bound to the starch. In the negative control (0 % of starch), Cgt was mainly found in the supernatant.

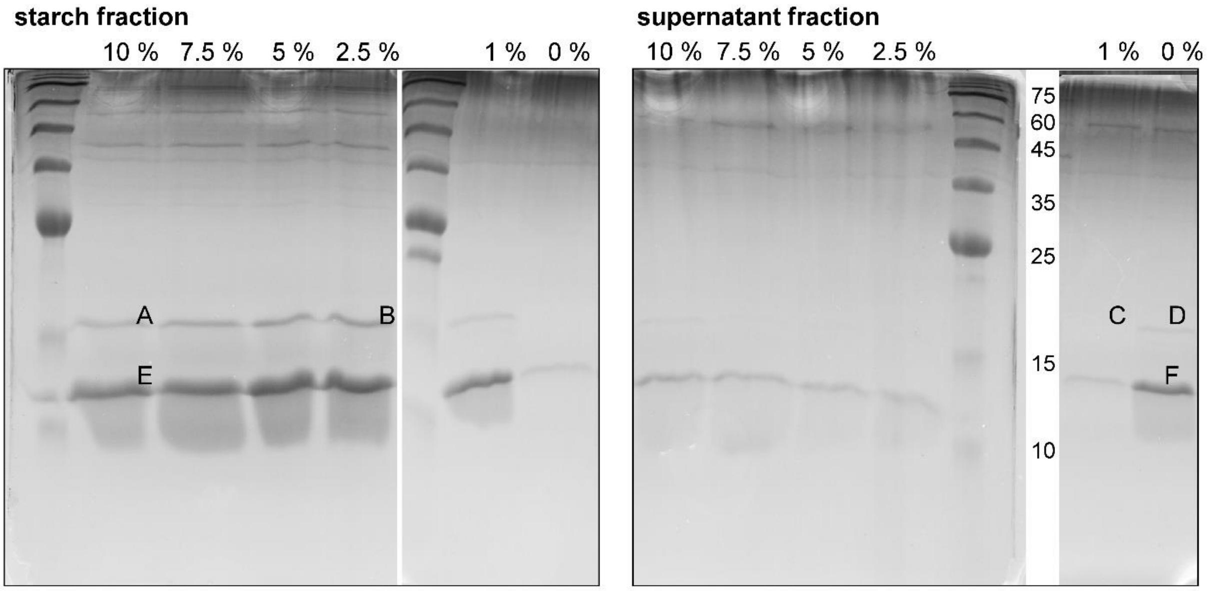

Fig. S2 Growth of the wild type of *Actinoplanes* sp. SE50/110 in minimal medium supplemented with different carbon sources. Shown are the medium cell dry weights and the standard deviation of at least three biological replicates (with the number of biological replicates n: n_{mal} = 5, n_{starch} = 5, $n_{C\text{-}Pur}$ = 3, n_{glc} = 3, n_{gal} = 4, n_{cel} = 4, n_{ara} = 5, n_{lac} = 3).

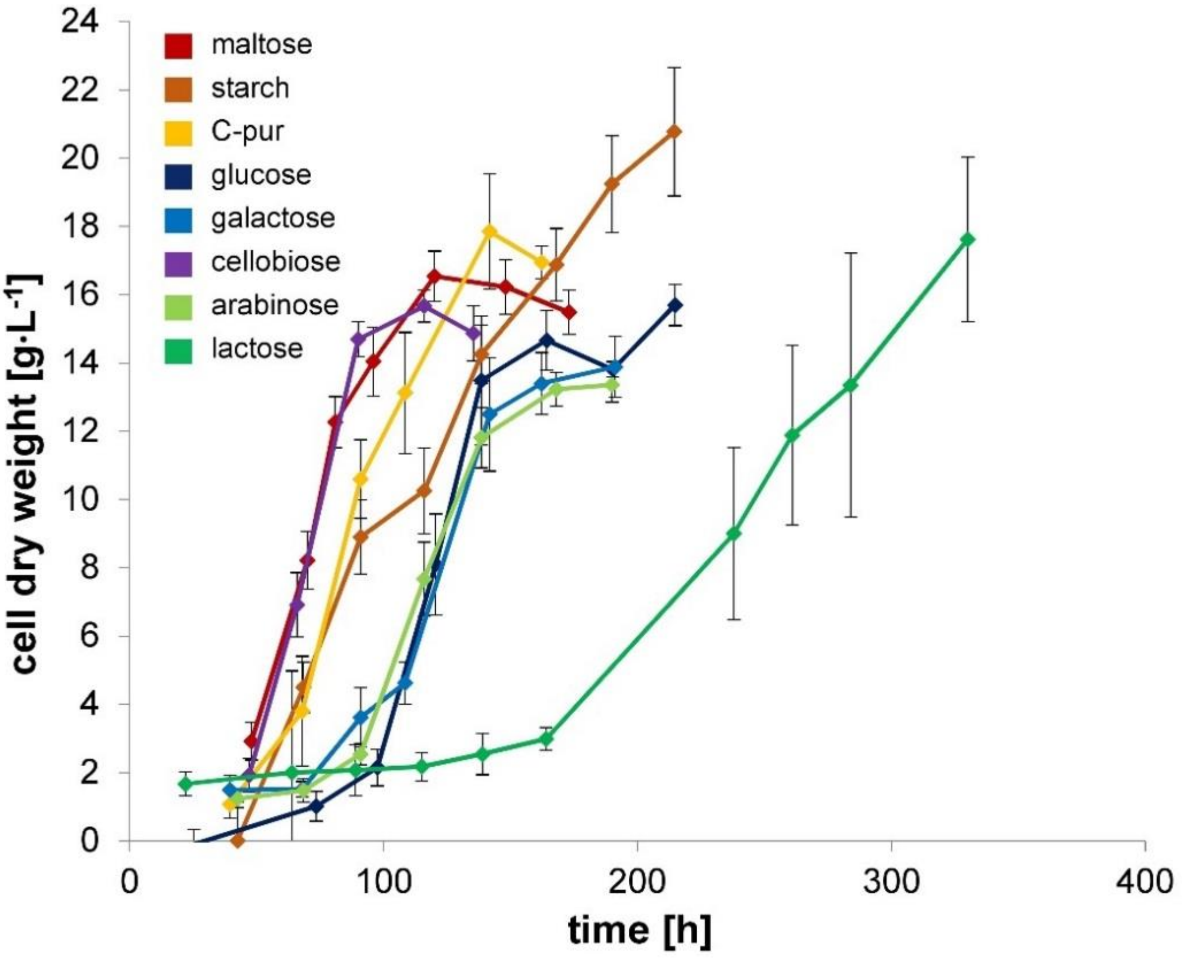

Fig. S3 Pre-screening experiments in the Biolog OmniLog Phenotypic Microarray System (Hayward, United States of America). Shown are the results of the phenotype microarray panels PM1 and PM2. Utilization of carbon sources is shown by the development of the tetrazolium dye G over time in each well according to the manufacturer’s instructions. The wild type is shown in black and the deletion mutant Δ*cgt* in violet.

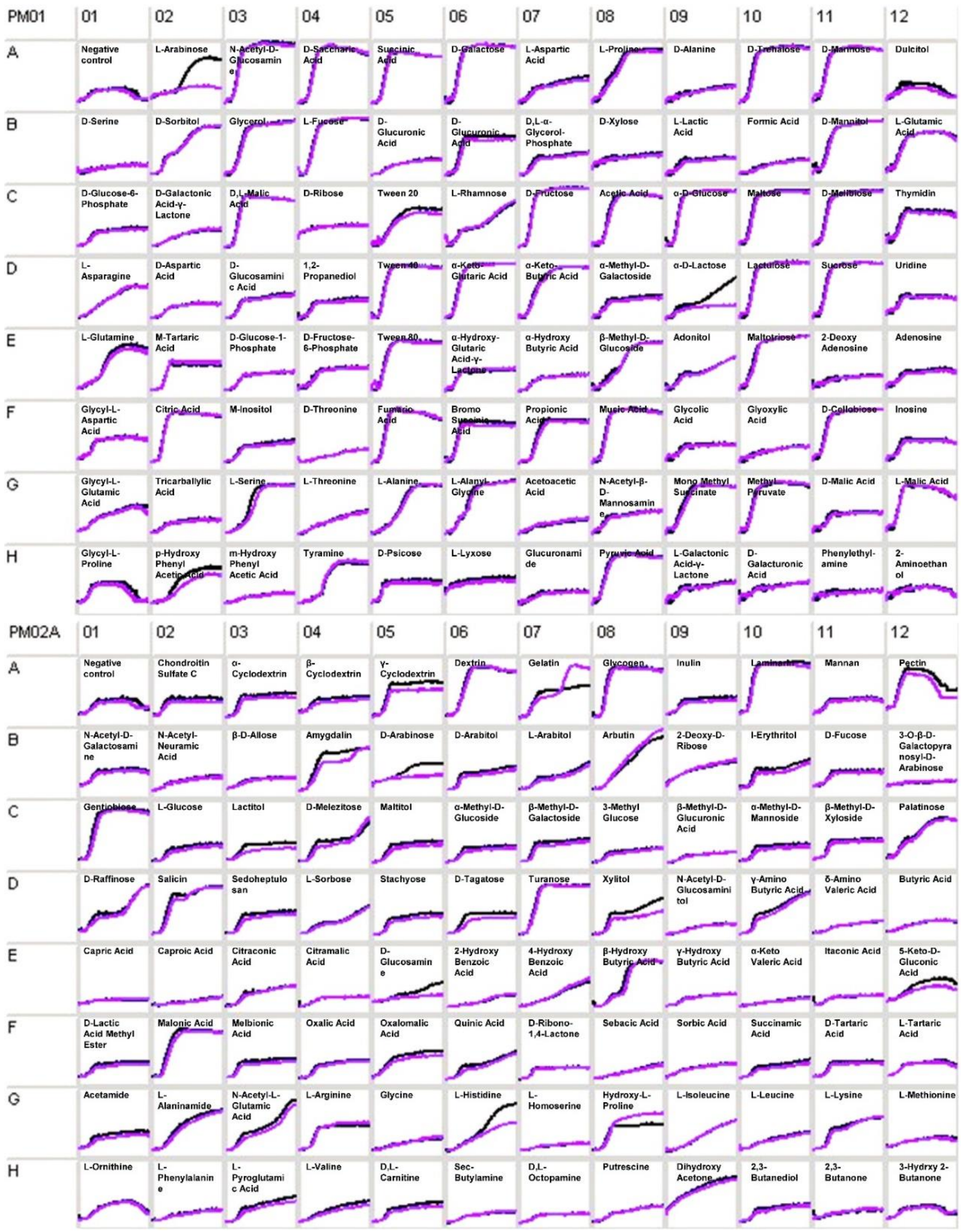

Fig. S4 Growth of the wild type and the deletion mutant Δ*cgt* of *Actinoplanes* sp. SE50/110 in minimal medium complemented with different carbon sources. Shown are the cell dry weights and the standard deviation over time (with the number of biological replicates n: wild type: n_{glc} = 3, n_{mal} = 5, n_{cel} = 4, n_{lac} = 3, n_{ara} = 5, n_{starch} = 5, Δ*cgt*: n_{glc} = 2, n_{mal} = 5, n_{cel} = 4, n_{lac} = 4, n_{ara} = 5, n_{starch} = 5).

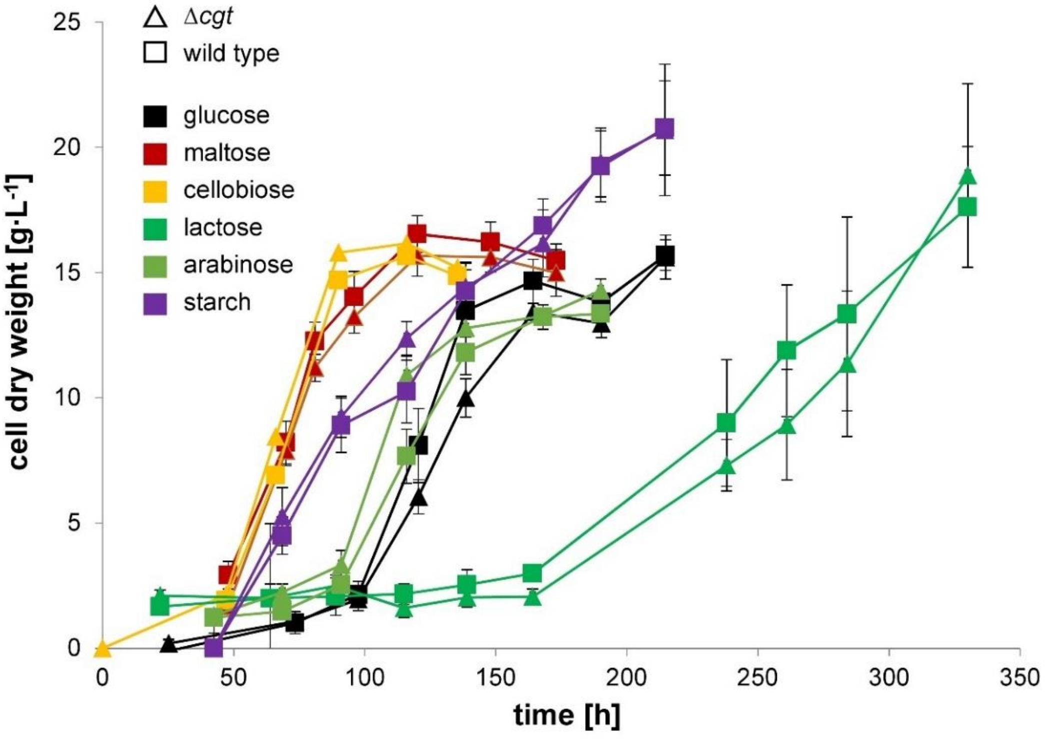

Fig. S5 Final cell dry weights obtained in cultivations of the wild type and the Δ*cgt* mutant in minimal media supplemented with six different carbon sources (compare to Fig. S4). The error bars denote the standard deviation.

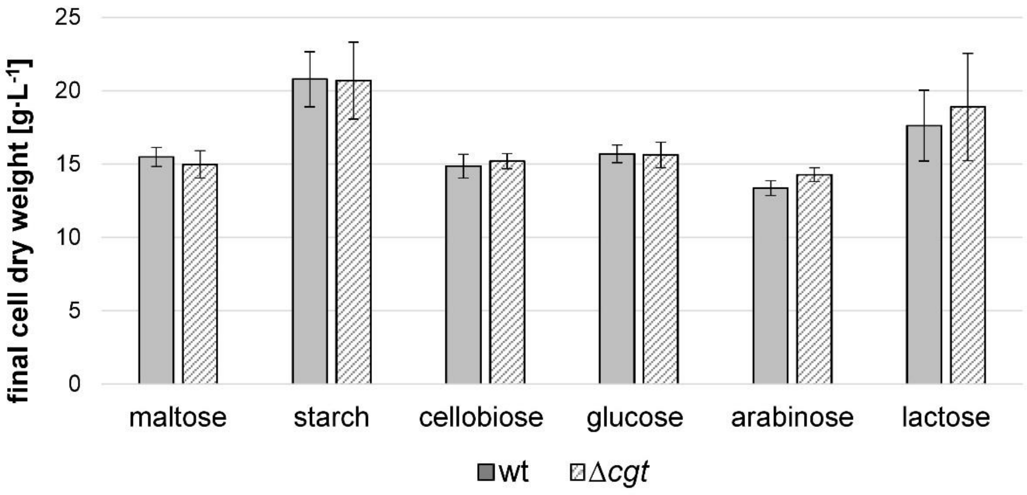

Fig S6 Growth of Δ*cgt* and the wild type under limited amounts of starch as carbon source. The medium was supplemented with 1 g·L^{-1}, 2 g·L^{-1}, 3 g·L^{-1}, 4 g·L^{-1} and 5 g·L^{-1} starch and the cultivation was performed in the RoboLector® system of m2p labs. Shown are the medium backscatter signals in a bar diagram and the standard deviation of at least three biological replicates. No growth restraints were observed for Δ*cgt.* Growth was even found to be significantly enhanced in a medium supplemented with 1 g·L^{-1} starch (p-value of a two-sided t-test: 0.006141, with the number of biological replicates n: n_{wt} = 3, $n_{\Delta cgt}$ = 4).

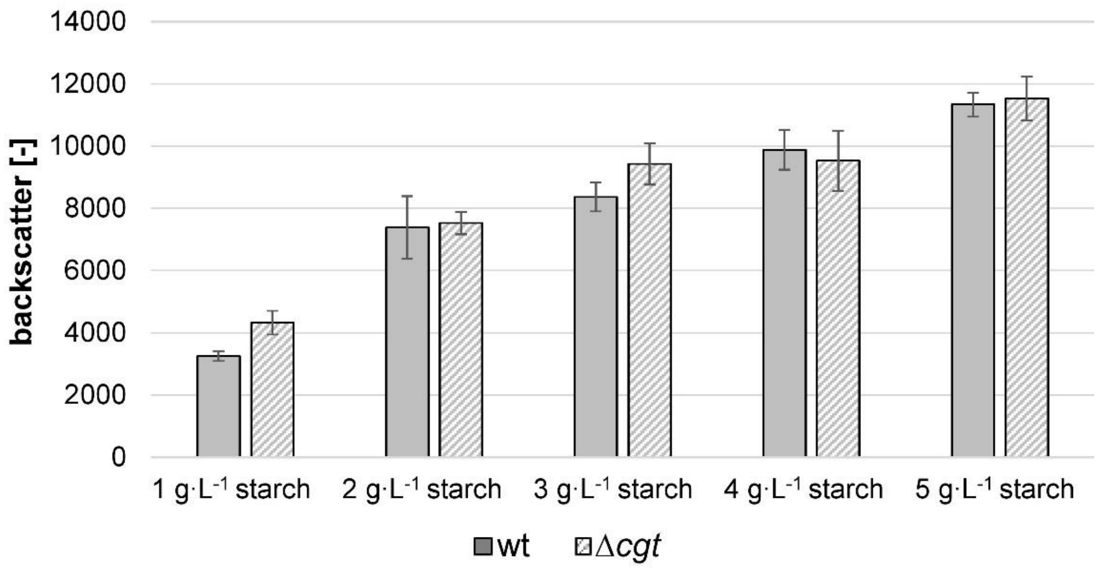

Fig. S7 A pH tolerance screening in solid culture. Upper dots correspond to the wild type; bottom dots correspond to the deletion mutant Δ*cgt* of *Actinoplanes* sp. SE50/110. Shown are droplets of a 7-step-dilution series of spores applied on SFM-agar plates with pH ranging from 4 to 11.

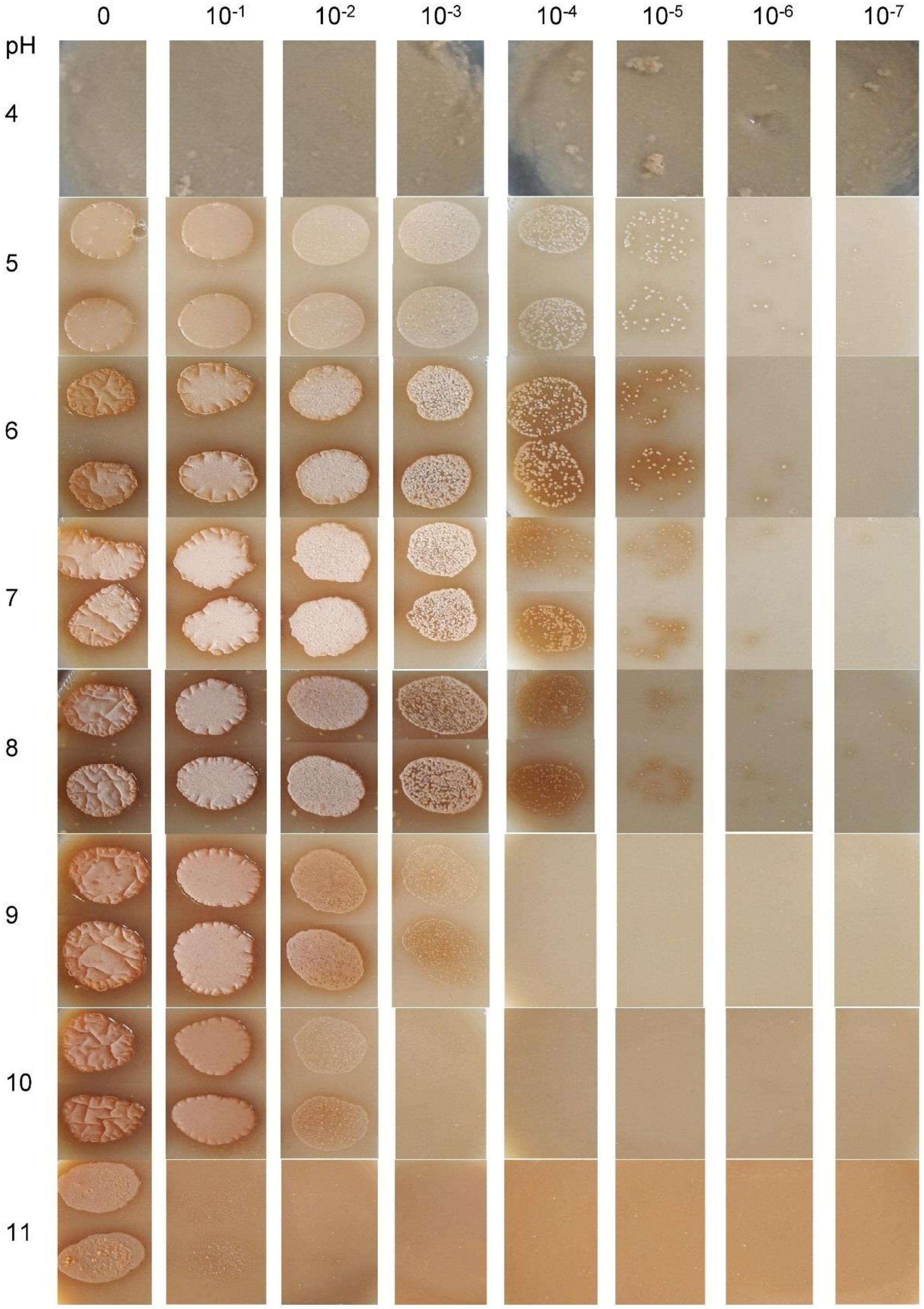

Fig. S8 A pH tolerance screening in an 1 mL reaction volume in the 48-well FlowerPlates in the RoboLector® system of m2p-labs. Growth of the wild type and Δ*cgt* in minimal medium adjusted to different pH ranging from pH 4 to 7 (with the number of biological replicates n: n_{wt} = 3, $n_{\Delta cgt}$ = 4).

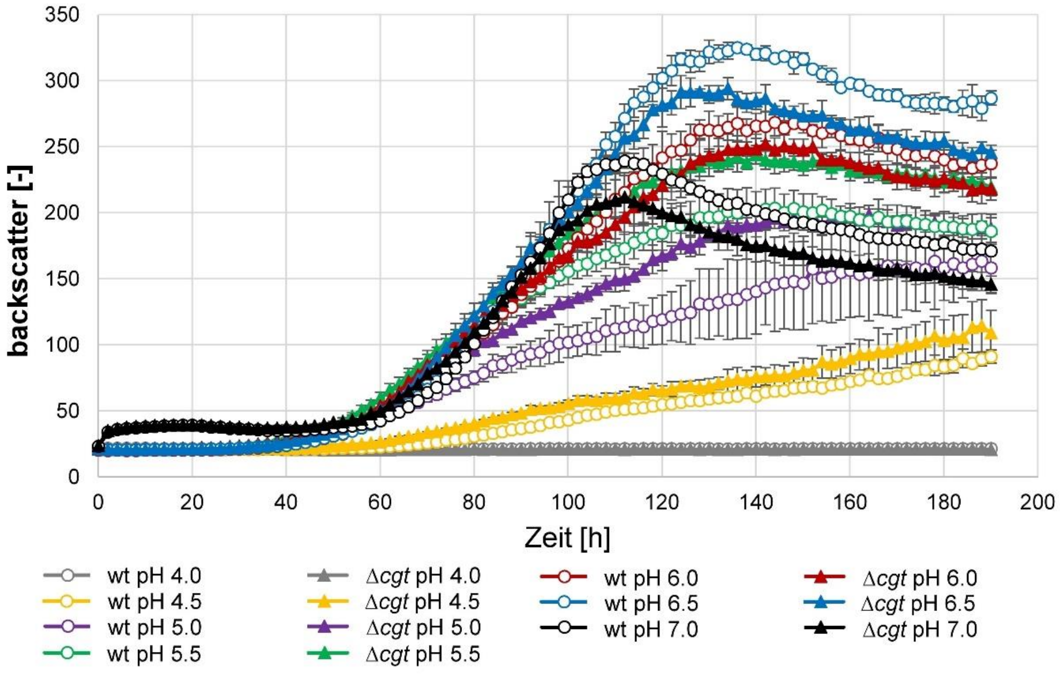

Fig. S9 Final cell dry weights of a pH screening experiment in maltose minimal medium. Wild type and Δ*cgt* mutant of *Actinoplanes* sp. SE50/110 were grown in an 1 mL reaction volume in a 48-well FlowerPlates in the RoboLector® system of m2p-labs. In pH ranging from 4 to 7, no significant differences in final cell dry weights were observed (tested by a two-sided t-test, with the number of biological replicates n: n_{wt} = 3, $n_{\Delta cgt}$ = 4, compare to Fig. S8).

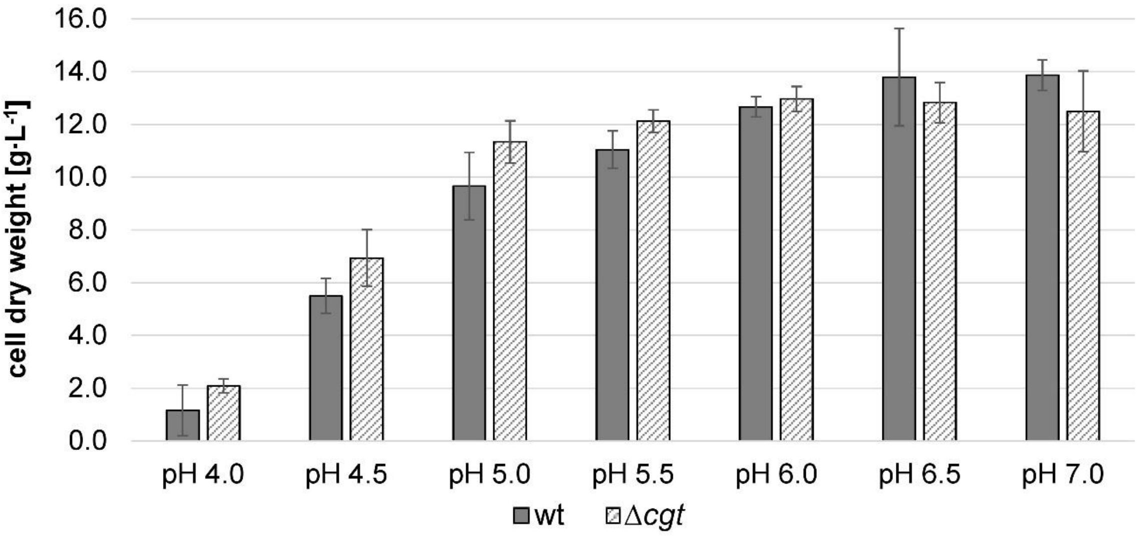

Fig. S10 An osmolality tolerance screening: Final cell dry weights in maltose minimal medium with maltose monohydrate concentrations ranging between 3.6 and 108.1 g·L^{-1}. No significant growth differences were observed (tested by a two-sided t-test, with the number of biological replicates n: n_{wt} = 3, $n_{\Delta cgt}$ = 4).

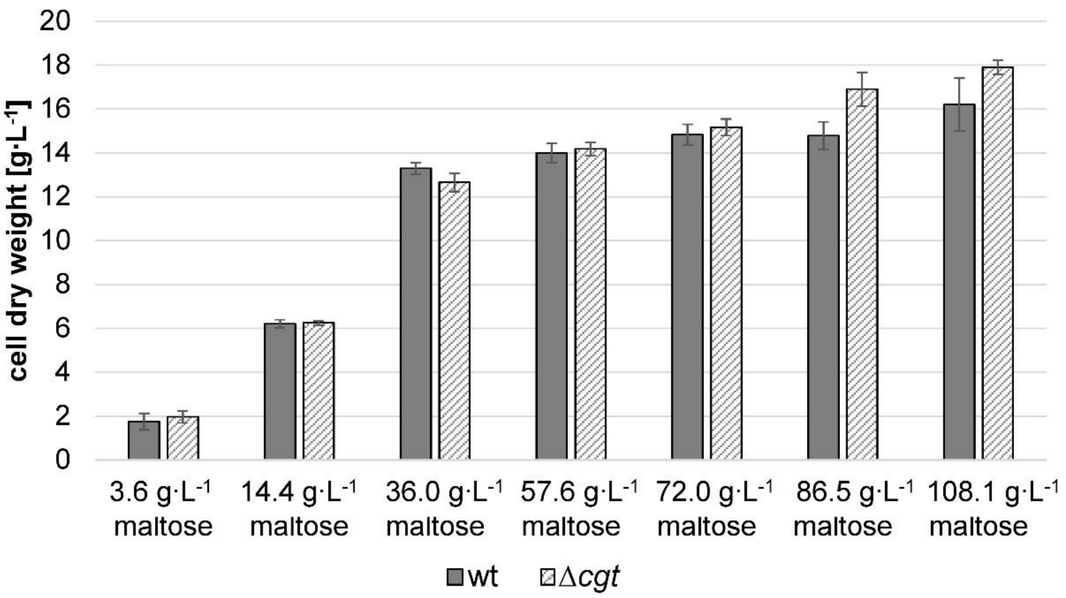

Fig. S11 An osmolality tolerance screening: Final cell dry weights of an osmolality-screening experiment in maltose minimal medium. The different osmolalities were achieved by addition of inositol in concentrations ranging from 0 mM to 280 mM. No significant growth differences between the wild type and Δ*cgt* were observed (tested by a two-sided t-test, with the number of biological replicates n: n_{wt} = 3, $n_{\Delta cgt}$ = 4).

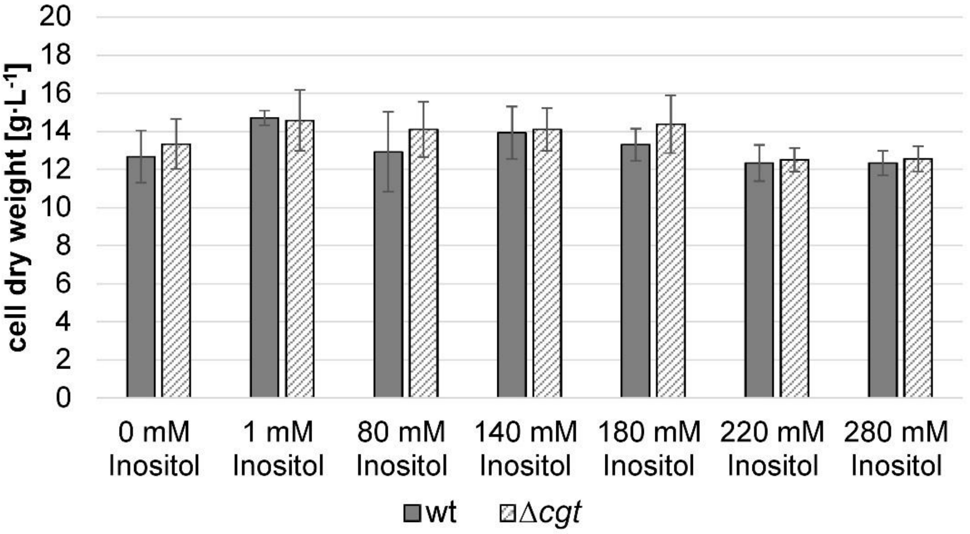

Fig. S12 Growth and acarbose production of *Actinoplanes* sp. SE50/110 wild type and Δ*cgt* mutant in the complex medium NBS supplemented with 11.0 $g \cdot L^{-1}$ maltose- respectively 10.0 $g \cdot L^{-1}$ glucose-monohydrate. No differential growth was observed. During growth phase, a significant increased acarbose concentration was measured in Δ*cgt* (significance of t-test after 49 h of cultivation: p-value = 0.006778, with the number of biological replicates n: $n_{wt\text{-}acb} = 3$, $n_{\Delta cgt\text{-}acb} = 3$, $n_{wt\text{-}cdwGlc} = 4$, $n_{\Delta cgt\text{-}cdwGlc} = 3$, $n_{wt\text{-}cdwMal} = 4$, $n_{\Delta cgt\text{-}cdwMal} = 4$).

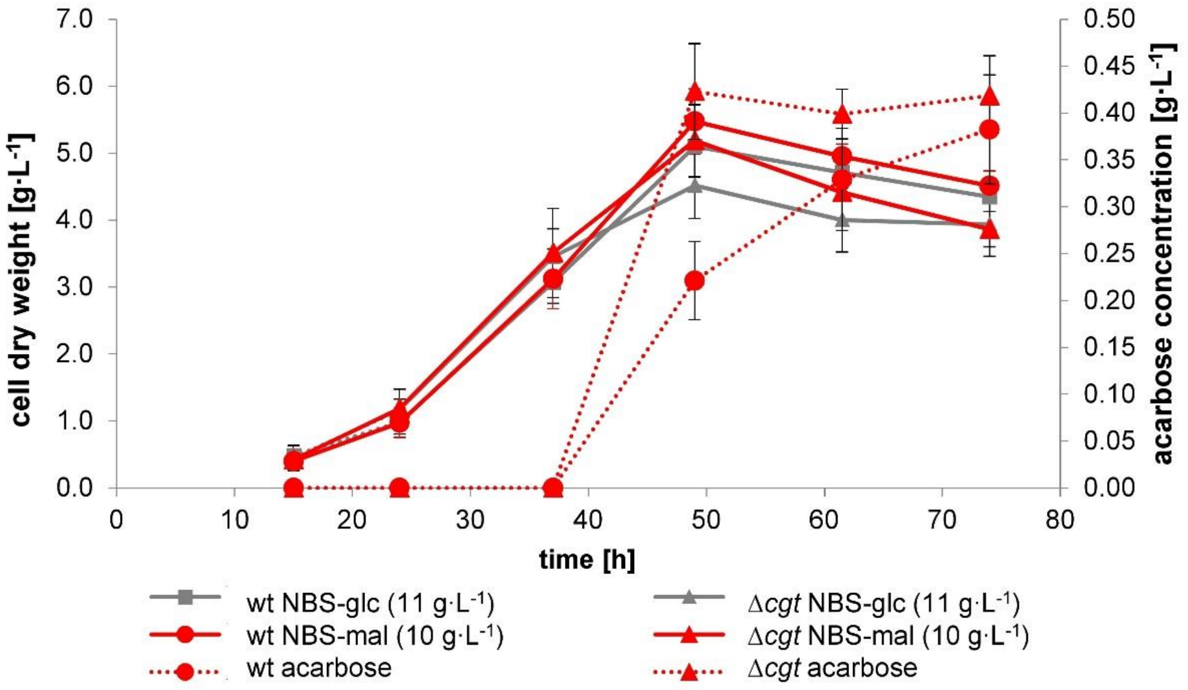

Fig. S13 Growth of *Actinoplanes* sp. SE50/110 wild type and Δ*cgt* mutant on maltose minimal medium. Shown are cell dry weights and acarbose concentrations of at least three biological replicates and the standard deviation (with n indicating the number of biological replicates).

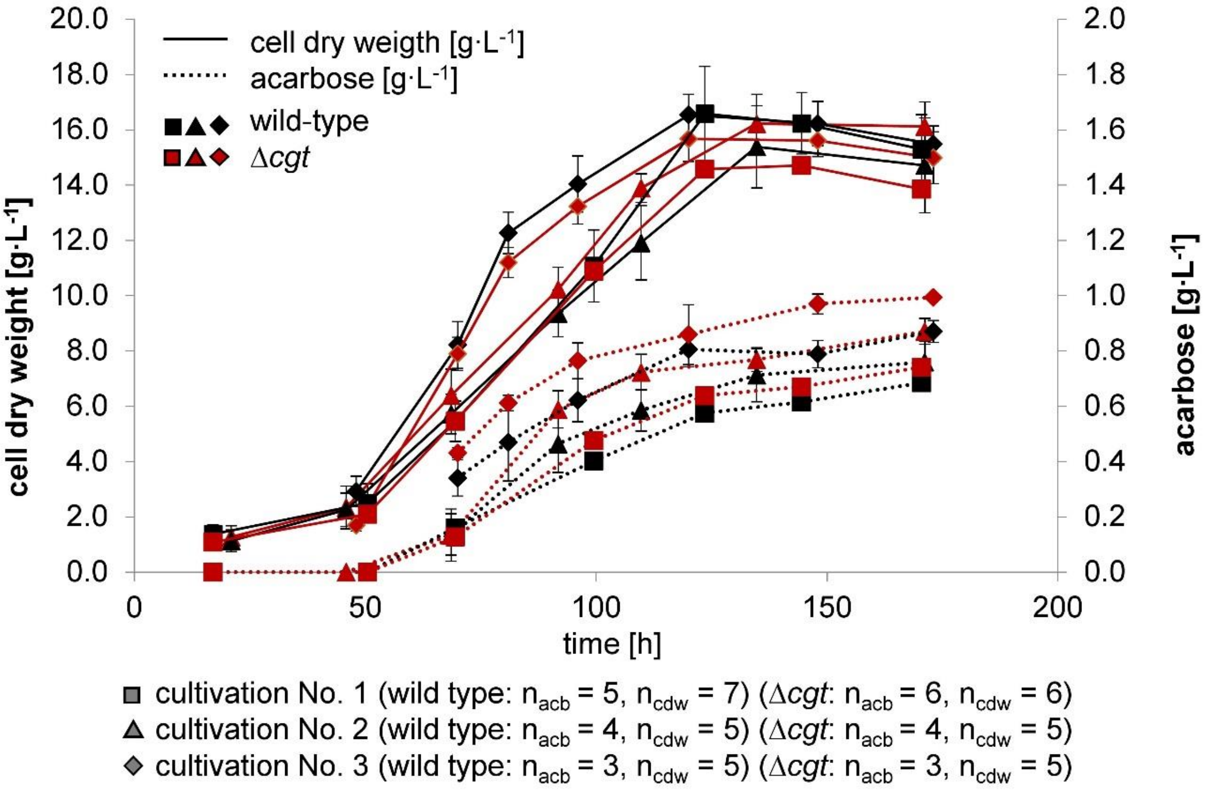

Fig. S14 Final yield coefficient of acarbose with reference to the cell dry weight shown in a box plot (created by the Interactive Dotplot tool (Weissgerber et al. 2017)). In three independent cultivations in maltose-minimal medium (No. 1-3), the Δ*cgt* mutant displays an enhanced acarbose producing phenotype compared to the wild-type (wt).

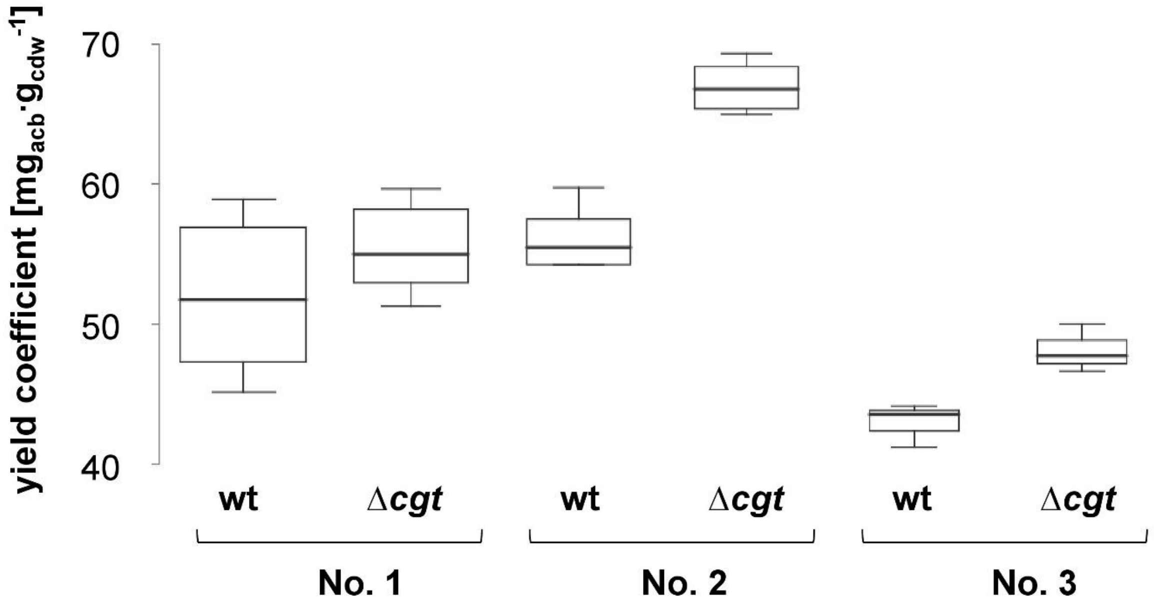

Table S1 MALDI-TOF-MS analysis of excised SDS-PAGE bands. Beside Cgt (ACSP50_5024), another small extracellular protein of unknown function (ACSP50_6253) and a transposase (ACSP50_0220) were identified.

**Number of excised band**	**Results of Mascot Search**		
**(compare to Fig. S1)**	**matches to**	**score**	**expect**
A	ACSP50_6253	87	3.3e-05
B	ACSP50_0220	47	0.91
C	ACSP50_6253	103	8.3e-07
D	ACSP50_6253	88	2.7e-05
E	ACSP50_5024	72	0.00095
F	ACSP50_5024	84	6.6e-05

Table S2 Tabular summary of the screening experiments. The final cell dry weights, the final acarbose concentrations, the pH and osmolality of different minimal media used in this work for screening of the osmolality and the pH[1].

	maltose x1H_2O ($g \cdot L^{-1}$)	glucose x1H_2O ($g \cdot L^{-1}$)	inositol (mM)	osmolality ($mOsmol \cdot kg^{-1}$)	pH	final cell dry weights ($g \cdot L^{-1}$)		final acarbose concentration ($g \cdot L^{-1}$)		
						wild type	Δ*cgt*	wild type	Δ*cgt*	significance (p-value)
**pH Screening**	72.06	-	-	629.0	4.0	1.17 ±0.95	2.09 ±0.26	not detectable		-
	72.06	-	-	630.0	4.5	5.50 ±0.66	6.94 ±1.07	0.27 ±0.18	0.38 ±0.05	*0.02216
	72.06	-	-	606.0	5.0	9.67 ±1.28	11.34 ±0.80	0.47 ±0.003	0.65 ±0.03	* 0.00136
	72.06	-	-	587.0	5.5	11.04 ±0.71	12.13 ±0.42	0.66 ±0.03	0.80 ±0.02	* 0.00124
	72.06	-	-	569.0	6.0	12.67 ±0.38	12.97 ±0.47	0.97 ±0.17	0.89 ±0.01	-
	72.06	-	-	569.0	6.5	13.79±1.84	12.83 ±0.76	1.14 ±0.13	1.06 ±0.04	-
	72.06	-	-	563.0	7.0	13.86 ±0.57	12.50 ±1.53	0.94 ±0.10	0.85 ±0.06	-
**Osmo-Screening 1**	3,6	-	-	323.5	6.5	1.75 ±0.38	1.97 ±0.28	not detectable		-
	14.41	-	-	361.5	6.4	6.21 ±0.19	6.25 ±0.10	not detectable		-
	36.03	-	-	420.5	6.4	13.29 ±0.26	12.66 ±0.41	0.33 ±0.04	0.39 ±0.08	-
	57.65	-	-	485.0	6.4	14.00 ±0.45	14.19 ±0.30	0.49 ±0.10	0.61 ±0.08	-
	72.06	-	-	531.0	6.4	14.83 ±0.47	15.17 ±0.38	0.58 ±0.05	0.65 ±0.06	-
	86.47	-	-	605.0	6.4	14.79 ±0.63	16.91 ±0.77	0.67 ±0.04	0.69 ±0.01	-
	108.09	-	-	681.0	6.3	16.21 ±1.20	17.91 ±0.33	0.56 ±0.03	0.66 ±0.02	* 0.01432
**Osmo-Screening 2**	20	-	0	388.5	6.4	11.88 ±0.22	12.25 ±0.25	not detectable		-
	20	-	1	406.5	6.4	12.83 ±0.47	12.75 ±0.98	not detectable		-
	20	-	80	469.5	6.4	12.75 ±0.45	12.06 ±0.95	not detectable		-
	20	-	140	550.0	6.4	15.13 ±0.25	13.16 ±0.75	not detectable		-
	20	-	180	580.0	6.4	14.63 ±0.45	14.03 ±1.15	not detectable		-
	20	-	220	610.3	6.4	14.79 ±0.44	13.13 ±1.76	not detectable		-
	20	-	280	695.0	6.3	15.33 ±0.51	14.00 ±1.45	not detectable		-
**NBS-Glc**	0	10	0	190	6,9	4.35 ±0.22	3.93 ± 0.47	acarbose is not main component		
**NBS-Mal**	11	-	0	159	6.9	4.51 ± 0.22	3.87 ± 0.27	0.38 ±0.06	0.42 ±0.04	*0.006778

1 Different osmolalities in the media used for pH screening are caused by addition of correcting agents.

Table S3 Tabular summary of the growth experiments under acarbose producing conditions. The final acarbose concentrations, the final cell dry weights and the number of replicates *n* of three independent cultivations of Δ*cgt* and the wild type in maltose minimal medium (compare to Figs. S13 and S14).

		**No.1**	**No.2**	**No.3**
**acarbose (g·L^{-1})**	WT	0.76 (+/- 0.07)	0.87 (+/- 0.04)	0.69 (+/- 0.02)
	n	5	4	3
	Δ*cgt*	0.88 (+/- 0.05)	0.99 (+/- 0.01))	0.74 (+/- 0.03
	n	6	4	3
**%**		116.6	114.2	108.3
**cell dry weight (g·L$^{-1)}$**	WT	14.72 (+/- 1.71)	15.49 (+/- 0.65)	15.30 (+/- 1.25)
	n	7	5	5
	Δ*cgt*	16.12 (+/- 0.89)	14.99 (+/- 0.94)	13.85 (+/- 1.46)
	n	6	5	5
**%**		109.5	96.8	90.5
